# Deciphering Interplay between *Salmonella* Invasion Effectors

**DOI:** 10.1371/journal.ppat.1000037

**Published:** 2008-04-04

**Authors:** Robert J. Cain, Richard D. Hayward, Vassilis Koronakis

**Affiliations:** University of Cambridge, Department of Pathology, Cambridge, United Kingdom; Tufts University School of Medicine, United States of America

## Abstract

Bacterial pathogens have evolved a specialized type III secretion system (T3SS) to translocate virulence effector proteins directly into eukaryotic target cells. *Salmonellae* deploy effectors that trigger localized actin reorganization to force their own entry into non-phagocytic host cells. Six effectors (SipC, SipA, SopE/2, SopB, SptP) can individually manipulate actin dynamics at the plasma membrane, which acts as a ‘signaling hub’ during *Salmonella* invasion. The extent of crosstalk between these spatially coincident effectors remains unknown. Here we describe *trans* and *cis* binary entry effector interplay (BENEFIT) screens that systematically examine functional associations between effectors following their delivery into the host cell. The results reveal extensive ordered synergistic and antagonistic relationships and their relative potency, and illuminate an unexpectedly sophisticated signaling network evolved through longstanding pathogen–host interaction.

## Author Summary

Critical to the onset of *Salmonella* infection is the ability of bacteria to force their own entry (‘invade’) into intestinal cells of their mammalian host from where they replicate, spread and cause damage. To achieve this invasion, *Salmonella* deliver a cocktail of proteins directly into host target cells. These proteins override host cell communications and remodel cell structure, tricking the normally dormant cells into engulfing the invaders. Although we are beginning to understand the functions of each delivered protein, little is known about how their activities are coordinated. Here we describe new techniques that systematically examine the interplay between the delivered bacterial proteins within the host cell. The results illuminate an unexpectedly complex network of interrelated relationships that must be precisely coordinated to promote bacterial invasion. The data provide new insights into how this important pathogen triggers invasion of host cells during infection.

## Introduction

Many bacterial pathogens employ type III secretion systems (T3SSs) to deliver virulence effector proteins directly into eukaryotic host cells [1]. An essential early T3SS-dependent step in *Salmonella* pathogenesis is bacterial invasion of non-phagocytic intestinal epithelial cells, an event that can be modelled using cultured cells [2]. Invading bacteria deliver effectors that induce actin-rich membrane ruffles, which drive pathogen internalization into a membrane-bound vacuole where they subsequently survive and replicate [3].

**Figure 1:**
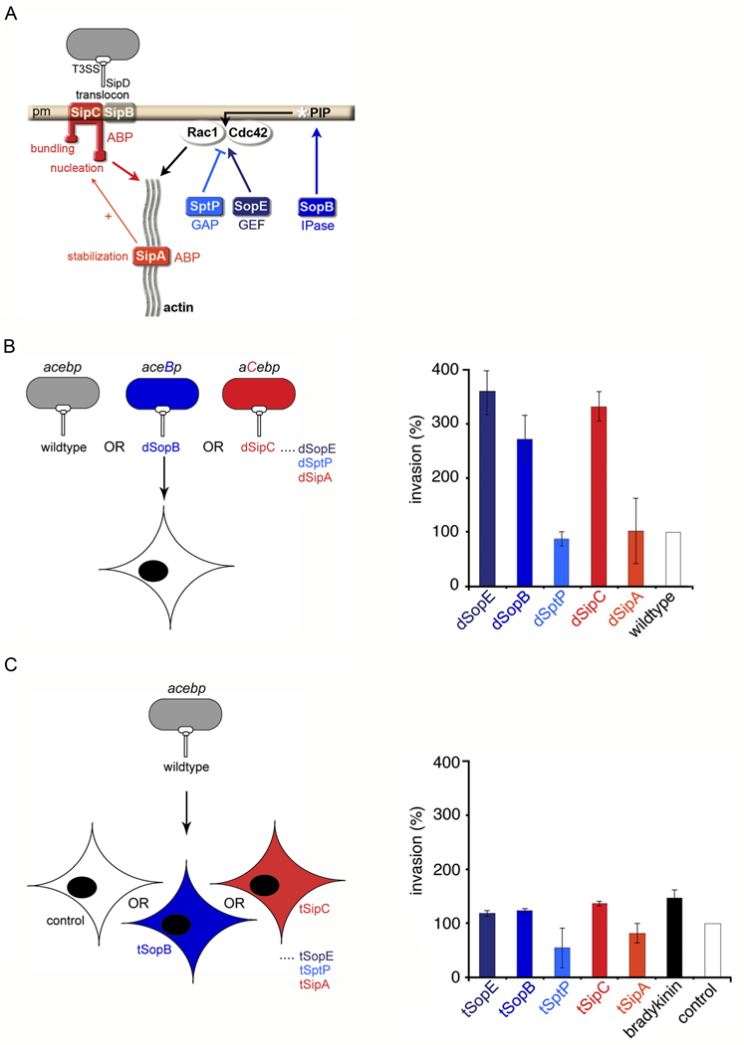
Only T3SS-delivered effectors influence bacterial invasion rate. A. *Salmonella* effectors that subvert host cytoskeletal dynamics. Effectors are delivered into the host cell via the type III secretion system (T3SS). Effector delivery requires *Salmonella* invasion proteins SipB and SipC, which form a plasma membrane-integral translocon likely linked to the T3SS by SipD. Two delivered effector Sips are actin-binding proteins (ABPs) that modulate actin dynamics: SipC nucleates actin polymerization and cross-links (bundles) actin filaments (F-actin) at the cell plasma membrane (pm), activities stimulated by SipA, which independently binds F-actin and inhibits filament depolymerization. Three further effectors are delivered into the cell via a SipB-SipC-dependent mechanism: the GDP-GTP exchange factor (GEF) SopE (or ubiquitous SopE2) activates Cdc42 and Rac1 Rho-family GTPases; the inositol polyphosphatase (IPase) SopB indirectly activates these GTPases and RhoG via inositol phosphate (PIP) hydrolysis; the antagonistic GTPase activating activity (GAP) and tyrosine phosphatase activities of SptP inactivate signaling after bacterial entry. B. Left: Schematic illustrating infection of cultured cells by wild-type or effector-augmented *S.typhimurium* strains. Wild-type bacteria endogenously express, secrete and deliver *sipA, sipC, sopE, sopB* and *sptP* (abbreviated to *acebp*). Effector-augmented strains each express, secrete and deliver mildly increased levels of an individual plasmid-encoded effector in the wild-type background [enhanced effector shown in capitals, *e.g. aceB*p (dSopB) and *aC*ebp (dSipC) produce increased levels of SopB and SipC, respectively]. Right: Graph comparing relative cell invasion rates of wild-type and effector-augmented (denoted d-effector) *S.typhimurium* strains. Invasion was compared to wild-type after 60 min (assigned as 100%). Results are mean±SEM of 4 independent experiments each performed in triplicate. C. Left: Schematic illustrating infection of effector-transfected cells by *S.typhimurium*. Wild-type bacteria endogenously express, secrete and deliver *sipA, sipC, sopE, sopB* and *sptP* (abbreviated to *acebp*). Cultured cells were transfected with individual effectors prior to infection (denoted t-effector). Right: Graph comparing invasion of mock transfected (control), effector-transfected (t-effector) or bradykinin-treated cells by wild-type *S.typhimurium*. Invasion was compared to wild-type after 60 min (assigned as 100%). Results are mean±SEM of 4 independent experiments each performed in triplicate.

Effectors are delivered into the target cell via a cholesterol-binding plasma membrane-integral translocon comprising SipB and SipC, which is likely linked to the T3SS by SipD [4],[5]. Six delivered effectors manipulate the target cell actin cytoskeleton (summarized in **Figure 1A**). Two *Salmonella* actin-binding proteins, SipC and SipA, control actin dynamics directly [6]. In addition to its role in effector delivery, discrete SipC domains nucleate actin polymerization and bundle actin filaments (F-actin) [7]. Both these SipC-directed activities are stimulated by SipA [8], which itself binds and stabilizes F-actin and suppresses actin turnover by host ADF/cofilin and gelsolin [9],[10]. Further effectors stimulate Rho-family GTPase signaling to induce cytoskeletal and nuclear responses [11]. The guanine nucleotide exchange factor mimic SopE (or ubiquitous SopE2) activates Cdc42 and Rac1 GTPases directly [12]–[14], whereas the inositol polyphosphatase SopB/SigD stimulates Cdc42, Rac-1 and the cellular RhoG SH3-containing guanine nucleotide exchange factor (SGEF) through induced phosphoinositide fluxes [12],[15],[16]. After invasion, Rho GTPase up regulation is antagonized by SptP, a GTPase activating protein (GAP) mimic and tyrosine phosphatase [17].

Discrete bacterial surface proteins of *Listeria* and *Yersinia* induce internalization by hijacking host receptor-mediated endocytosis [18]. In contrast, individual *Salmonella* effectors are insufficient to promote bacterial internalization, although SopE, SopB and SipC do elicit generalized cell-wide cytoskeletal reorganization and membrane protrusions when expressed individually in cells [8],[14],[19],[20]. It is likely that delivered effectors must therefore act in concert to induce productive actin rearrangements rapidly and specifically beneath invading *Salmonellae* without compromising target cell viability [6],[11].

We have previously demonstrated that *Salmonella* entry effectors localize to the target cell plasma membrane both when expressed individually in cultured cells and after delivery via the bacterial T3SS, and consequently we proposed the plasma membrane as a critical interface for *Salmonella* effector action [19]. We next explored how the activities of these spatially co-incident effectors might potentially be coordinated to trigger actin rearrangements. Here we describe systematic experimental screens that illuminate the extent, potency and hierarchy of interplay between *Salmonella* effectors and their host targets within cultured cells.

## Results

### Only effectors delivered by the *Salmonella* T3SS promote invasion

To investigate the subcellular localization of effectors delivered by the invasion-associated *Salmonella* T3SS [19], we generated a bank of wild-type (WT) *S.typhimurium* SL1344 strains each additionally expressing an individual plasmid-encoded epitope-tagged entry effector. This allowed the expression, secretion and delivery of an individual effector to be specifically enhanced (1.5–3.3-fold WT) in a WT background (described in detail in Materials and Methods and [19]). Hereafter, these WT strains engineered to deliver increased doses of particular effectors are termed ‘effector-augmented strains’, and the enhanced effector as ‘d-*effector*’.

When cultured cells were infected with the effector-augmented strains (**Figure 1B** **, left**), we observed that a significant increase in bacterial invasion rate occurred with the dSopE, dSopB or dSipC strains (**Figure 1B** **, right** ∼300%; *i.e.* an approximately 3-fold increase compared to WT normalized to 100%). Each of these strains secrete the other entry effectors at WT levels [19], but since SipC additionally functions in effector translocation in complex with SipB [7], we additionally investigated the effect of enhancing the levels of the other translocation-associated effectors SipB and SipD (**Figure S1A**). In contrast to the dSipC strain, no significant increase in invasion rate occurred upon infection with effector-augmented dSipB or dSipD strains (dSipB 120±15.8%, dSipD 80±14%; **Figure S1B**). Nevertheless, consistent with previous observations [5],[21], SipB localized to both the plasma membrane and the bacterial surface, whereas SipD could only be weakly detected on the surface of some (∼10%) internalized bacteria (**Figure S1C**). Therefore, the increased invasiveness of the dSipC strain likely arises from SipC effector function rather than any unrelated effect on effector translocation.

As *S.typhimurium* mutants lacking *sptP* and WT are equally invasive [17], the observed rate increases associated with the dSopE, dSopB and dSipC strains likely reflect the stimulatory action of the augmented effector rather than decreased delivery of the signal-suppressing effector SptP. Indeed, levels of delivered SopE and SopB in WT, an *sptP* mutant and the dSptP strain are indistinguishable (D. Humphreys, unpublished). In addition, SopE, SopB or SipC are each able to stimulate actin polymerization in cultured cells, although alone are insufficient to trigger bacterial entry [6],[11]. *Salmonella sopE* or *sopB* deletion mutants exhibit only a modest decrease in invasion, whereas *sipC* null mutants are non-invasive, as SipC is not only an effector but also a translocator of other effectors [4],[16],[22]. These combined data therefore strongly support linkage between the delivered dose of stimulatory effectors, the resulting extent of actin polymerization and *Salmonella* invasion rate.

However, when cultured cells were transfected with effectors (denoted ‘t-*effector*’) and infected with WT *S.typhimurium* (**Figure 1C** **, left**), only mild rate increases (∼120% *i.e.* an approximately 0.2-fold increase when compared to WT normalized to 100%) were evident. A comparable effect was observed when control Cdc42-dependent actin polymerization and macropinocytosis were induced with bradykinin (**Figure 1C** **, right**). tSptP and tSipB conferred a limited resistance to invasion (54±37% and 56±22%, respectively), consistent with their ability to counteract cytoskeletal and nuclear responses and to disrupt cellular endomembranes, respectively (**Figure 1C** **, right**; **Figure S1D**; **Figure S1E**; [17],[23]). Additionally, we verified that transfectants remained viable and that effectors were expressed and localized correctly (**Figure S2A**). Indeed, the concentration of each t-effector in the plasma membrane exceeded that of the corresponding d-effectors in infected cells (**Figure S2B**). Thus, although tSopE, tSopB and tSipC all trigger dramatic cytoskeletal reorganization [19], unlike delivered effectors (**Figure 1B** **, right**), these transfected effectors are unable to significantly influence invasion rate, either because the effectors distribute along the plasma membrane throughout the cell, rather than at specific pathogen contact sites as with the T3SS-delivered effectors, or because their premature activity inhibits WT entry. Together these data reveal that the spatio-temporal context of effector activity seems critical, as only cytoskeletal rearrangements induced by delivered stimulatory effectors can significantly increase bacterial invasion rate.

### SipC stimulates invasion independently of cellular Rho-family GTPases

**Figure 2:**
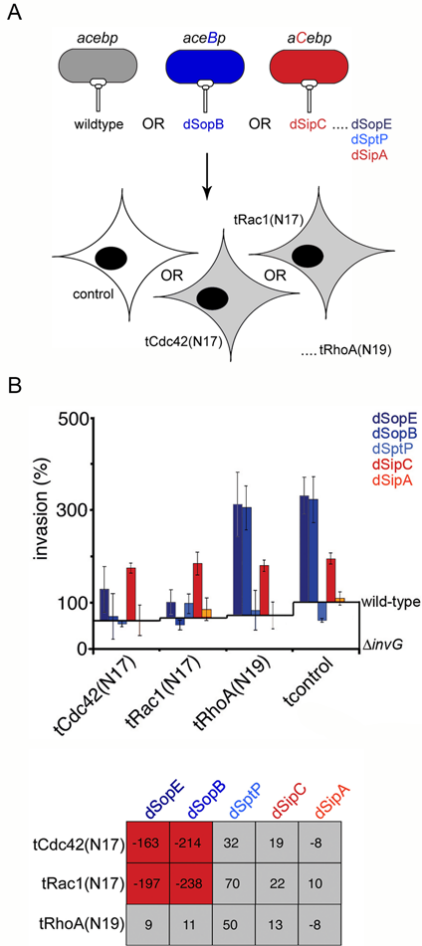
SipC-mediated invasion occurs independently of cellular Rho GTPases. A. Schematic illustrating the infection of cells expressing dominant negative Rho-family GTPases by wild-type or effector-augmented *S.typhimurium* strains. Wild-type bacteria endogenously express, secrete and deliver *sipA, sipC, sopE, sopB* and *sptP* (abbreviated to *acebp*). Effector-augmented strains each express, secrete and deliver mildly increased levels of an individual plasmid-encoded effector in the wild-type background [enhanced effector shown in capitals, *e.g. aceB*p (dSopB) and *aC*ebp (dSipC) produce increased levels of SopB and SipC, respectively]. Cultured cells were transfected with dominant negative Rho-family GTPases [Cdc42(N17), Rac1(N17), RhoA(N19)] prior to infection (denoted t-dominant negative GTPase). B. Cultured fibroblasts were transfected with dominant negative Rho-family GTPases [tCdc42(N17), tRac1(N17), tRhoA(N19)] prior to infection with wild-type or effector-augmented (d-effector) *S.typhimurium* strains. Invasion rates after 60 min were compared to wild-type (assigned as 100%). Results are mean±SEM of 4 independent experiments each performed in triplicate. Baselines ‘wild-type’ and ‘Δ*invG*’ denote WT *S.typhimurium* SL1344 and *S.typhimurium* Δ*invG* (T3SS deficient) invasion in each transfectant background, respectively. Table shows differences in invasion rates (%) after correction. Shading denotes a significant decrease (red) or no significant change (grey) in invasion rates (Mann Whitney U p<0.05).

To determine whether the enhanced invasion rates of the dSopE, dSopB and dSipC strains reflect specific effector-induced stimulation of defined cellular signaling pathways, we next investigated the consequence of transfecting cells with dominant negative Rho-family GTPases prior to infection with WT *Salmonella* or each of the effector-augmented strains (**Figure 2A**). As expected, tCdc42(N17), tRac1(N17) and tRhoA(N19) expression suppressed WT *S.typhimurium* invasion by ∼40%, confirming Rho-family GTPases as important cellular targets of delivered *Salmonella* effectors (**Figure 2B**; [14],[24]). When tCdc42(N17) or tRac1(N17) transfectants were infected with the dSopE or dSopB strains, the previously observed increases in invasion rate significantly diminished (*e.g.* tCdc42(N17):dSopE –163%; tRac1(N17) −238%), yet remained unaltered by tRhoA(N19) expression. Conversely, none of the expressed dominant negatives suppressed the enhanced invasion rate of the dSipC strain (*e.g.* tCdc42(N17) +19%; tRhoA(N19) +13%), indicating that SipC activity is Rho-family GTPase independent (**Figure 2B**). Furthermore, unlike the WT, the invasion rate of the dSipC strain was equivalent in each dominant negative background (*i.e.* in **Figure 2B**, compare constant dSipC invasion rate to decreases in WT rate in the dominant negative backgrounds), suggesting that augmenting SipC delivery can compensate for the reduction in SopE/SopB-dependent stimulation of Cdc42/Rac1-dependent signaling. This is consistent with the ability of SipC to induce actin reorganization directly [7]. These data illustrate that three entry effectors cooperate in the WT strain to stimulate parallel Cdc42/Rac1-dependent (SopE, SopB) and –independent (SipC) invasion pathways, the relative contribution of which can be modulated by the comparative levels of each effector delivered.

### Ordered effector interplay revealed by *trans* BENEFIT screening

**Figure 3:**
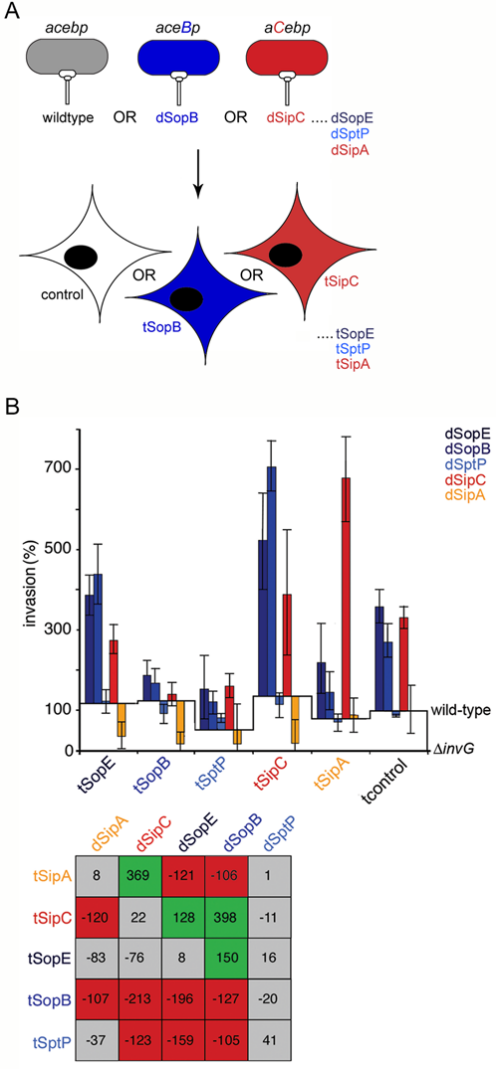
Ordered effector interplay revealed by *trans* BENEFIT screening. A. Schematic illustrating *trans* BENEFIT screening (the infection of cells expressing individual entry effectors by wild-type or effector-augmented *S.typhimurium* strains). Wild-type bacteria endogenously express, secrete and deliver *sipA, sipC, sopE, sopB* and *sptP* (abbreviated to *acebp*). Effector-augmented strains each express, secrete and deliver mildly increased levels of an individual plasmid-encoded effector in the wild-type background [enhanced effector shown in capitals, *e.g. aceB*p (dSopB) and *aC*ebp (dSipC) produce increased levels of SopB and SipC, respectively]. Cultured cells were transfected with individual entry effectors (denoted t-effector) prior to infection. B. Cultured fibroblasts were transfected (t) with individual effectors prior to infection with wild-type or effector-augmented (d-effector) *S.typhimurium* strains. Invasion rates after 60 min were compared to wild-type (assigned as 100%). Results are mean±SEM of 4 independent experiments each performed in triplicate. Baselines ‘wild-type’ and ‘Δ*invG*’ denote *S.typhimurium* SL1344 and *S.typhimurium* Δ*invG* (T3SS deficient) invasion in each transfectant background, respectively. Table shows differences in invasion rates (%) after correction. Shading denotes a significant increase (green), significant decrease (red) or no significant change (grey) in invasion (Mann Whitney U p<0.05).

Based on this transfection-infection approach, we next designed binary entry effector interplay (BENEFIT) screens to assess potential cross talk between pairs of effectors. In the first ‘*trans’* BENEFIT screen, we investigated the effects of expressing individual bacterial effectors rather than dominant negative derivatives of host proteins in cells prior to infection with WT *Salmonella* or the effector-augmented strains (**Figure 3A**). We conservatively defined functional interplay between effector A (‘d-*effector*’; augmented levels of which are delivered via the T3SS in a WT background) and effector B (‘t-*effector*’ pre-expressed in the target cell by transient transfection) to be an increase (or decrease) in rate of at least one-fold of WT after correction for WT *Salmonella* invasion of cells expressing effector B (**Figure 1C** **, right**), and invasion of control cells by WT *Salmonella* expressing augmented levels of effector A (**Figure 1B** **, right**). This threshold is significantly different from the controls (Mann Whitney U p<0.05).

The results of this screen were striking, revealing reproducible changes in *Salmonella* invasion rates (**Figure 3B** **;** **Figure S3**). Our criteria identified four synergistic relationships. Two effector combinations induced mild increases in invasion rates (dSopE:tSipC +128%; dSopB:tSopE +150%), whereas two further pairings stimulated even more prominent changes (dSipC:tSipA +369%; dSopB:tSipC +398% *i.e.* the invasion rate was ∼4-fold greater than WT entry into control cells). Direct observation of infected cells in parallel revealed dramatic actin reorganization induced by these pairings (**Figure S4**), the morphology of which often reflected a combination of their reported activities [8],[14],[19]. These rate and phenotypic variations were not a simple function of augmenting effector dose, as no significant effects were observed using homotypic effector combinations under identical conditions (*i.e.* dSopE:tSopE +8%; dSipC:tSipC +22%; **Figure 3B** and **Figure S2C**). Furthermore, all these effects were apparently also dependent on ordered effector activity, as inverse pairing resulted in inhibition of bacterial invasion (*e.g.* dSipC:tSopE −76%; dSipA:tSipC –120%). An extended screen including the dSipB and dSipD strains confirmed that unlike SipC, which is involved in both effector delivery and actin reorganization [4],[7],[22], SipB and SipD neither functionally contribute to invasion nor engage in synergy with any delivered effectors (**Figure S3A; Figure S3B**).

Significant inhibitory effects were also evident (**Figure 3B**). Premature expression of either SopB or SptP in cells suppressed subsequent infection by several *Salmonella* strains, in particular dSopE (tSopB, −196%; tSptP, −159%), dSopB (−127%, −105%) or dSipC (−213%, −123%), and as with effector synergy, this functional antagonism was reversed (*e.g.* tSipC:dSopB +398%; tSopE:dSopB +150%) or annulled (*e.g.* tSipC:dSptP −11%; tSopE:dSptP +16%) using inverse pairings. Notably, although both tSptP and tSipB impede WT invasion (tSptP 54±37%; tSipB 56±22%), the effector-augmented strains were not significantly inhibited by tSipB expression (**Figure S3B**). The inhibitory effects reflect true variations in invasion rate rather than indiscriminate changes due to differential effector-induced cellular toxicity, as effector transfection generated no significant alteration in the number or viability of target cells (**Figure S2A**), a conclusion reinforced further by the direct observation of internalized bacteria (**Figure S4**).

While *trans* BENEFIT screening permitted the sequence of effector activity to be dictated experimentally, an associated limitation is the imposed effector concentration imbalance, as the effector levels in transfected cells exceed those delivered by the bacteria. However, when effector concentrations were assessed following infection and transfection by quantitative immunoblotting, analysis revealed the difference between transfected and delivered effector concentrations was less than one order of magnitude (3–8 fold excess in transfectants; **Figure S2B**). Fractionation of transfected cells following infection with the effector-augmented strains also demonstrated that both the transfected and delivered effectors remained co-localized in the plasma membrane and cytoskeletal fractions, as when expressed or delivered in isolation (**Figure S2C**; [19]). Coupled to the fact that a ‘dominant negative’ phenotype is induced only by tSopB and tSptP, these control data further suggest that disordered effector activity rather than non-specific effector concentration or mislocalization likely accounts for this antagonism. Thus, the *trans* BENEFIT data suggest that extensive functional interplay does occur between delivered *Salmonella* effectors.

### Sip-Sop synergy revealed by *cis* BENEFIT screening

**Figure 4:**
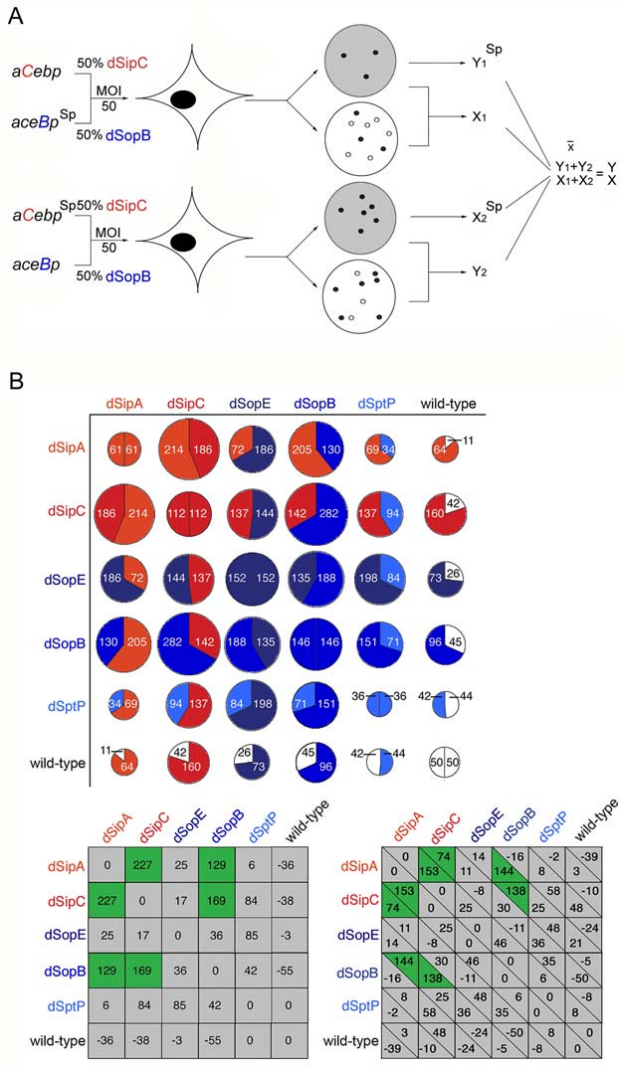
Sip-Sop synergy revealed by *cis* BENEFIT screening. A. Schematic illustrating *cis* BENEFIT screening. Pair-wise combinations of effector-augmented (d-effector) *S.typhimurium* strains [enhanced effector shown in capitals, *e.g. aceB*p (dSopB) and *aC*ebp (dSipC) produce increased levels of SopB and SipC, respectively] were mixed 50∶50 (overall MOI 50), where one strain additionally carries spectinomycin resistance (Sp^R^). The mixed inoculum is used to infect cultured cells, and a reciprocal infection performed in parallel in which the opposing strain is spectinomycin resistant. After infection (60 min), extracellular bacteria are killed with gentamicin and infected cell lysate dilutions replica plated on LB agar (depicted white) and LB containing spectinomycin (grey). Overall invasion rate and invasion of the spectinomycin resistant strain are calculated by scoring colony-forming units. The invasion rate of the strain lacking the marker is calculated by subtracting invasion of the spectinomycin resistant strain from the overall value (X_1_, Y_2_). To correct for the mild influence of spectinomycin on invasion efficiency, invasion rates of individual strains are averaged with the parallel experiment *i.e.* [X_1_+X_2_^Sp^]÷2 = X and [Y_1_+Y_2_^Sp^]÷2 = Y. Each pair of infections were performed 4 times in triplicate. B. *S.typhimurium* SL1344 or effector-augmented (d-effector) strains were mixed pair wise (50∶50; MOI 50) for infection. Invasion of each strain was assessed using selectable markers after 60 min (Figure 4A). Results are the mean of four independent experiments each performed in triplicate Upper: Pie charts depict total invasion by each combination (size; combined %) and relative contribution of each strain (division; %). Lower: Tables show difference (%) in total invasion rate (left) and relative invasion rate of each strain (right) after correction. Shading denotes a significant increase (green) or no significant change (grey) in invasion rate (Mann Whitney U p<0.05).

We next modified the BENEFIT screen to eliminate the imposed ordered effector activity and the concentration bias resulting from transfection. In this ‘*cis*’ screen, cultured cells were infected with pairwise mixtures of effector-augmented strains, in combination with each other or WT *S.typhimurium* (**Figure 4A**).

At first, an identical multiplicity of infection to the *trans* screen was used, except that each infection mixture comprised an equal proportion of two distinct effector-augmented *S.typhimurium* strains. An antibiotic marker was introduced into one strain to allow the relative invasion efficiency of each to subsequently be calculated. Since the selection conferred a marginal competitive disadvantage during mixed infection when compared to the isogenic non-resistant strain (∼−5%), each infection was performed in duplicate, allowing the strain carrying the marker to be alternated. The mean invasion efficiency of each strain could then be calculated and controlled for the marker-induced variation (**Figure 4A**). We initially confirmed that 50∶50 mixtures of isogenic input strains were recovered at an equivalent output ratio, *i.e.* strains that differed only with respect to the resistance marker did not compete with each other and were recovered with equivalent frequency from the infected cells [**Figure 4B**; *e.g.* mixed WT:WT invasion rate was 100% to which component strains contribute equivalently (50%∶50%)], and that the enhanced invasion rates observed with the dSopE, dSopB and dSipC strains (**Figure 1B** **, right**) were recapitulated during mixed infection [**Figure 4B**; *e.g.* the invasion rate of a mixed dSopE:dSopE strain input remains ∼3-fold WT (304%) to which component strains contribute equivalently (152%∶152%)].

Having experimentally verified these important assumptions, we next performed a complete *cis* BENEFIT screen using heterologous mixtures of effector-augmented WT strains. As previously, we conservatively defined functional interplay between effector A (‘d-*effector*’) and effector B (‘d-*effector*’; delivered by a separate strain) to be reflected by an increase (or decrease) in invasion rate of at least one-fold of WT from the change recorded when WT *Salmonella* expressing augmented levels of either effector A or B enter cells independently (**Figure 1B** **, right**). Such changes are statistically significantly different from the controls (Mann Whitney U p<0.05).

As might be expected, the resulting variations in invasion rate were lower in magnitude than in the *trans* BENEFIT screen, again illustrating that effector concentration is related to invasion rate, provided that host targets (*e.g.* actin, Rho GTPases, phosphoinositides) remain in excess. Two significant synergistic relationships were immediately evident (**Figure 4B**, dSipA:dSipC, +227%; dSipC:dSopB, +169%) that independently corroborated the most significant data from the *trans* BENEFIT screen (**Figure 3** **;** **Figure S3**), and an additional potential synergy also emerged, albeit nearer to the significance threshold (**Figure 4B**, dSipA:dSopB, +129%). Distinct synergistic classes could also be defined; ‘mutual’ where both strains benefit from their association [*e.g.* both the dSipC (+74%) and the dSipA (+153%) strains gain from a mixed dSipC:dSipA infection], or ‘selfish’ in which one strain exploits its partner [*e.g.* only the dSipA strain (+144%) benefits from a mixed dSipA:dSopB infection, whereas invasion of the dSopB strain is unchanged or marginally compromised (−16%)]. In contrast to the *trans* BENEFIT screen, no significant antagonistic effects were observed (**Figure 4B**). This is consistent with the view that inhibition arises from transfecting the cells with effectors prior to invasion that subsequently interfere with the ordered activities of bacterially-delivered effectors.

Co-infection with the effector-augmented strains never enhanced WT *S.typhimurium* invasion. Indeed, when mixed with the dSopE or dSipA strains the WT was appreciably but not significantly disadvantaged, as in mixed WT:dSipA and WT:dSopE infections, the WT invasion rate was reduced from the expected 50% to 11% and 26%, respectively (**Figure 4B**). Enhanced expression of SipC also conferred a mild selective advantage over the WT, as invasion of the dSipC strain increased from the expected 112% to 160% when co-infected with WT (**Figure 4B**). These data illustrate that one strain does not simply passively assist the invasion of any partner. Rather, the increased invasion conferred on any particular strain relates to the concentration and activity of the effectors within the target cell delivered by the strain itself or its co-infecting partner. To confirm this further, we performed additional assays in which the initial infection mixtures were artificially biased (90:10/10:90) to favour one or other of the strains. Under these conditions, the previously observed synergy was abolished as the recovered output reproducibly mirrored the composition of the input mixture (**Figure S5**). A threshold level of both delivered effectors is therefore necessary to drive the observed synergy.

### SipC-SopB synergy is driven by SipC-dependent clustering of plasma membrane phosphatidylinositol-4,5-bisphosphate

Our BENEFIT screening suggested that unexpectedly sophisticated interplay occurs between multiple delivered *Salmonella* entry effectors. Next, we further investigated one of the previously unrecognized relationships using complementary genetic, biochemical and cell biology techniques. We selected SipC-SopB synergy for further analysis, as this was apparently a dominant association identified by both the *trans* and *cis* BENEFIT screens.

**Figure 5:**
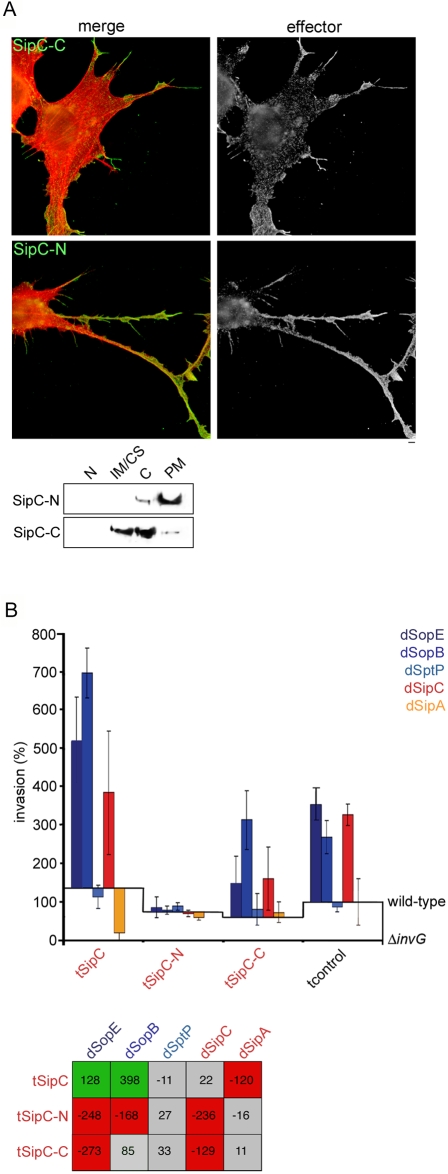
Membrane localization of the C-terminal SipC actin-nucleation domain is required for synergy with SopB. A. Localization of SipC derivatives expressed in cultured cells. Upper: Immunofluorescence micrographs of fixed NIH3T3 cells transiently expressing SipC-C or SipC-N. Left column shows merged double immunofluorescence (merge) of cells stained 48 h after transfection with anti-FLAG IgG/AlexaFluor 488-conjugated anti-mouse IgG to localise SipC derivatives (green) and Texas Red-conjugated phalloidin to visualize F-actin (red). Effector channel alone is shown in greyscale for clarity (effector). Images are representative of >100 cells from three independent experiments. Scale bars, 2 µm. Lower: Cultured NIH3T3 cells were transiently transfected with expression vectors encoding SipC-C or SipC-N. After 48 h transfectants were mechanically fractionated, and each subfraction [nuclei (N), internal membranes/cytoskeleton (IM/CS), cytoplasm (C), plasma membrane (PM)] analyzed by immunoblotting with anti-SipC polyclonal antibody. B. Cultured fibroblasts were transfected (t) with SipC or derivatives SipC-N or SipC-C prior to infection with wild-type or effector-augmented (d-effector) *S.typhimurium* strains. Invasion rates after 60 min were compared to wild-type (assigned as 100%). Results are mean±SEM of 4 independent experiments each performed in triplicate. Baselines ‘wild-type’ and ‘Δ*invG*’ denote *S.typhimurium* SL1344 and *S.typhimurium* Δ*invG* (T3SS deficient) invasion in each transfectant background, respectively. Table shows differences in invasion rates (%) after correction. Shading denotes a significant increase (green), significant decrease (red) or no significant change (grey) in invasion (Mann Whitney U p<0.05).

Initially, we wished to establish whether known SipC and SopB activities were required for synergy. The SipC N-terminal domain (SipC-N; residues 1–120) directs actin filament bundling, whereas the C-terminal domain (SipC-C; 200–409) induces actin nucleation [7]. Correspondingly, tSipC-N and tSipC-C both reorganized actin when expressed in cultured cells (**Figure 5A**). tSipC-C localized to the leading cell edge where it induced lamellipodial and filopodial protrusions reminiscent of those generated by membrane-integral SipC [8],[19], yet was distributed between the cytoskeletal, cytosolic and plasma membrane fractions upon mechanical fractionation (**Figure 5A, SipC-C**). tSipC-N localized along induced hyper-elongated filopodia 30–60 µm in length, an activity similar to the cellular actin-bundling protein fascin [25], and predominantly to the plasma membrane fraction upon mechanical fractionation (**Figure 5A, SipC-N**). However, when cells expressing SipC-N or SipC-C were infected with effector-augmented *S.typhimurium* strains, SipC-SopB synergy was attenuated (**Figure 5B**). Indeed, unlike tSipC, tSipC-N significantly inhibited invasion by the dSopE, dSopB and dSipC strains (**Figure 5B**, *i.e.* compare dSopE:tSipC-N, −248%; dSopB:tSipC-N, −168%; dSipC:tSipC-N, −236% to dSopE:tSipC, +128%, dSopB:tSipC, +398%, dSipC:tSipC, +22%), as premature actin bundling is likely to intrinsically impede cytoskeletal plasticity required for bacterial invasion. tSipC-C also inhibited dSopE and dSipC invasion (**Figure 5B**, dSopE:tSipC-C, −273%; dSipC;tSipC-C, −129%), but could still apparently engage in limited synergy with the dSopB strain, albeit below our imposed significance threshold (dSopB:tSipC-C, +85%). These data suggest a potential link to C-terminal domain function, but additionally imply that plasma membrane localization of this region is important for efficient synergy with SopB, reaffirming the view that the plasma membrane is a critical interface for effector interplay [19].

**Figure 6:**
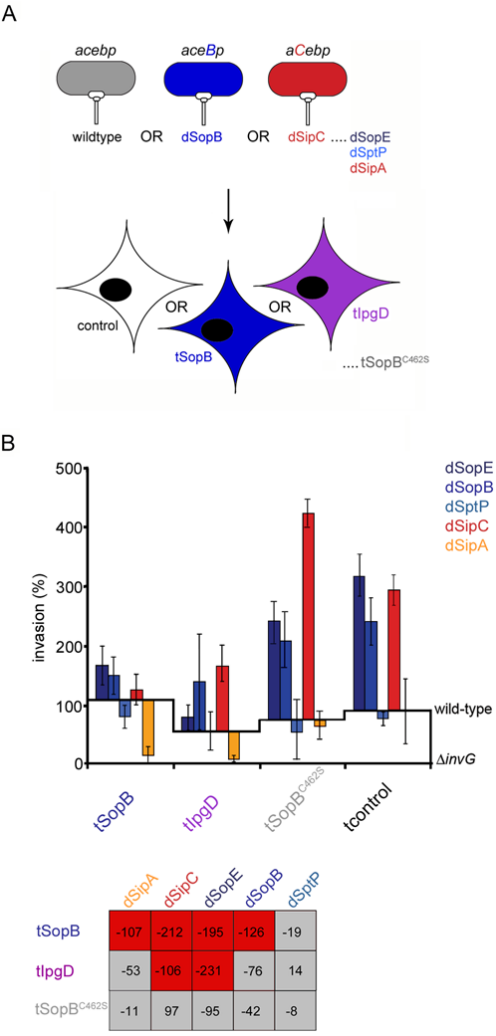
SopB inositol phosphatase activity is required for inhibition of *Salmonella* entry. A. Schematic illustrating the infection of cells expressing SopB, catalytically inactive SopB^C462S^ or the *Shigella* inositol phosphatase IpgD with wild-type or effector-augmented *S.typhimurium* strains. Wild-type bacteria endogenously express, secrete and deliver *sipA, sipC, sopE, sopB* and *sptP* (abbreviated to *acebp*). Effector-augmented strains each express, secrete and deliver mildly increased levels of an individual plasmid-encoded effector in the WT background [enhanced effector shown in capitals, *e.g. aceB*p (dSopB) and *aC*ebp (dSipC) produce increased levels of SopB and SipC, respectively]. Cultured cells were transfected with individual entry effectors or effector derivatives (denoted t-effector) prior to infection. B. Cultured fibroblasts were transfected (t) with SopB, a catalytically inactive SopB^C462S^ derivative, or the *Shigella* inositol phosphatase IpgD prior to infection with wild-type or effector-augmented (d-effector) *S.typhimurium* strains. Invasion rates after 60 min were compared to wild-type (assigned as 100%). Results are mean±SEM of 4 independent experiments each performed in triplicate. Baselines ‘wild-type’ and ‘Δ*invG*’ denote *S.typhimurium* SL1344 and *S.typhimurium invG* (T3SS deficient) invasion in each transfectant background, respectively. Table shows differences in invasion rates (%) after correction. Shading denotes a significant increase (green), significant decrease (red) or no significant change (grey) in invasion (Mann Whitney U p<0.05).

Next, we investigated whether SopB inositol phosphatase activity was required for SipC-SopB synergy by examining the effects of expressing the homologous invasion-associated inositol phosphatase *Shigella flexneri* IpgD [26] or a phosphatase-dead SopB derivative containing a C462S mutation in the active site [SopB^C462S^; [20]] prior to infection with the bank of effector-augmented *S.typhimurium* strains (**Figure 6A**). When pre-expressed in cells, tSopB is a ‘dominant-negative’ inhibitor of the dSipA, dSipC, dSopE and dSopB strains (**Figure 3B; Figure 6B**). This effect was recapitulated using tIpgD (dSipA:tIpgD, −53%; dSipC:tIpgD, −106%; dSopE:tIpgD, −231%; dSopB:tIpgD, −76%), although inhibition of the dSipA and dSopB strains was somewhat reduced. Nevertheless, this functional substitution suggested that inhibition is driven by inositol phosphatase activity rather than an unknown SopB-specific function. In clear support of this, significant inhibition was alleviated using phosphatase-dead tSopB^C462S^ (**Figure 6B**; dSipA:tSopB^C462S^, −11%; dSopE:tSopB^C462S^, −95%; dSopB:tSopB^C462S^, −42%; dSptP:tSopB^C462S^, −8%), and invasion of the dSipC strain specifically but mildly enhanced (dSipC:tSopB^C462S^, +97%).

**Figure 7:**
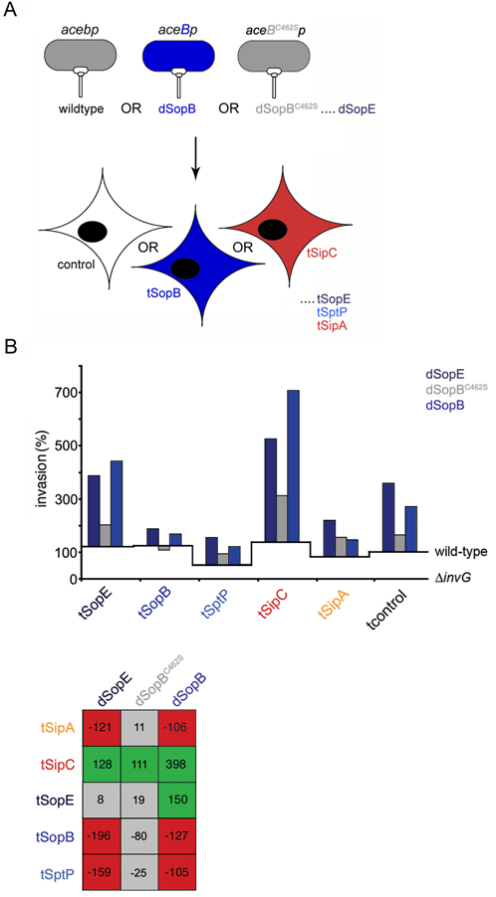
SopB inositol phosphatase activity is required for synergy with SipC during *Salmonella* entry. A. Schematic illustrating *trans* BENEFIT screening to investigate the role of SopB inositol phosphatase activity. Wild-type bacteria endogenously express, secrete and deliver *sipA, sipC, sopE, sopB* and *sptP* (abbreviated to *acebp*). Effector-augmented strains each express, secrete and deliver mildly increased levels of an individual plasmid-encoded effector in the wild-type background [enhanced effector shown in capitals, *e.g.* in this assay *aceB*p (dSopB), *aceB^C462S^p* (dSopB^C462S^) and *acEbp* (dSopE) that produce increased levels of SopB, catalytically inactive SopB^C462S^ or SopE, respectively]. Cultured cells were transfected with individual entry effectors (denoted t-effector) prior to infection. B. Cultured fibroblasts were transfected (t) with individual effectors prior to infection with wild-type or effector-augmented (d-effector) *S.typhimurium* strains. In this assay dSopE, dSopB and dSopB^C462S^ strains were examined. Invasion rates after 60 min were compared to wild-type (assigned as 100%). Results are typical of those obtained in two independent experiments each performed in triplicate. Baselines ‘wild-type’ and ‘Δ*invG*’ denote *S.typhimurium* SL1344 and *S.typhimurium* Δ*invG* (T3SS deficient) invasion in each transfectant background, respectively. Table shows differences in invasion rates (%) after correction. Shading denotes a significant increase (green), significant decrease (red) or no significant change (grey) in invasion (Mann Whitney U p<0.05).

To investigate the effect of SopB inositol phosphatase activity on SipC synergy during infection, we generated a *S.typhimurium* strain engineered to deliver increased levels of SopB^C462S^ in the WT background (dSopB^C462S^), and included this in a *trans* BENEFIT screen where effector-transfected cells were infected with dSopE, dSopB or dSopB^C462S^ strains (**Figure 7A**). Synergy between tSipC and dSopB^C462S^ was markedly attenuated to near the statistical threshold (**Figure 7B**, compare tSipC:dSopB, +298% to tSipC:dSopB^C462S^, +111%), whereas the weaker tSopE synergy was abolished (**Figure 7B**, compare tSopE:dSopB, +150% to tSopE:dSopB^C462S^, +19%). The latter is a recognized relationship mediated by cellular inositol phosphates [16]. Additionally, unlike the dSopB strain, dSopB^C462S^ invasion was not inhibited in cells expressing SipA, SopB or SptP (*e.g.* compare tSptP:dSopB, −105% to tSptP:dSopB^C462S^, −25%). These combined findings suggested that SopB inositol phosphatase activity contributes to synergy with membrane localized SipC.

**Figure 8:**
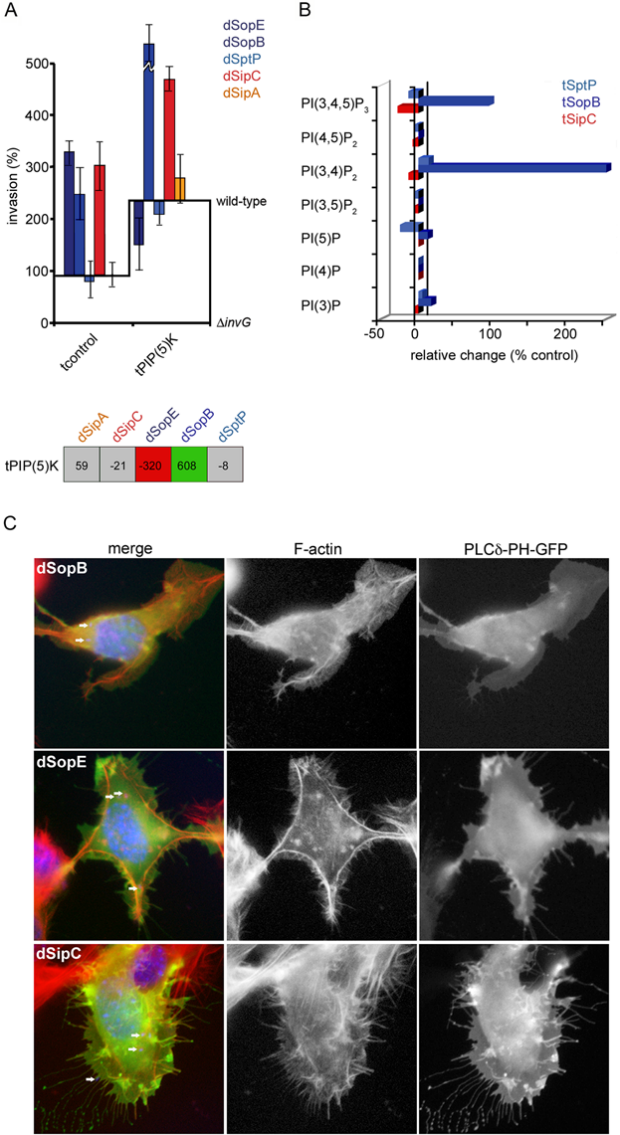
SipC-dependent relocalization of phosphatidylinositol-4,5-bisphosphate (PI_45_P2) drives synergy with SopB. A. Fibroblasts were transfected with cellular phosphatidylinositol-4-phosphate-5-kinase (tPIP(5)K) prior to infection with wild-type or effector-augmented (d-effector) *S.typhimurium* strains. Invasion rates after 60 min were compared to wild-type (assigned as 100%). Results are mean±SEM of 4 independent experiments each performed in triplicate. Baselines ‘wild-type’ and ‘Δ*invG*’ denote *S.typhimurium* SL1344 and *S.typhimurium* Δ*invG* (T3SS deficient) invasion in each transfectant background, respectively. Table shows differences in invasion rates (%) after correction. Shading denotes a significant increase (green), significant decrease (red) or no significant change (grey) in invasion (Mann Whitney U p<0.05). B. Graph showing relative change (compared to mock transfected control cells) in concentration of specific phosphatidylinositol species [PI(3,4,5)P_3_, phosphatidylinositol-3,4,5-trisphosphate; PI(4,5)P_2_, phosphatidylinositol-4,5-bisphosphate; PI(3,4)P_2_, phosphatidylinositol-3,4-bisphosphate; PI(3,5)P_2_, phosphatidylinositol-3,5-bisphosphate; PI(5)P, phosphatidylinositol-5-phosphate; PI(4)P, phosphatidylinositol-4-phosphate; PI(3)P, phosphatidylinositol-3-phosphate) in transfectants expressing SptP, SopB and SipC following HPLC analysis of radiolabelled fibroblasts. Data shown are representative of those obtained in two independent labelling experiments. C. Immunofluorescence micrographs of NIH3T3 cells expressing PLCδ-PH-GFP fixed after infection (60 min) with *S.typhimurium* strains engineered to express augmented levels of SopB, SopE or SipC. Left column shows merged triple immunofluorescence (merge) of cells stained with Texas Red-conjugated phalloidin to visualize F-actin (red) and DAPI to visualize cell nuclei and bacteria (blue, internalized bacteria indicated with arrows). GFP fluorescence was visualized directly. Actin (F-actin) and PLCδ-PH-GFP channels are also shown in greyscale for clarity. Images are representative of >100 cells from independent experiments. Scale bars, 2 µm.

SopB additionally aids invasion by cleaving plasma membrane phosphatidylinositol-4,5-bisphosphate [PI(4,5)P_2_] to promote membrane elasticity and vacuole formation [20]. To establish whether levels of PI(4,5)P_2_ influence invasion the augmented dSopB strain, we expressed phosphatidylinositol-4-phosphate-5-kinase [PIP(5)K], which catalyzes the formation of PI(4,5)P_2_, in cells prior to infection with the effector augmented *S.typhimurium* strains. Strikingly, excess PI(4,5)P_2_ stimulated WT *Salmonella* invasion more than two-fold (**Figure 8A**), and specifically and significantly enhanced dSopB strain invasion (**Figure 8A**; tPIP(5)K:dSopB, +608%), while inhibiting the dSopE strain (**Figure 8A**; tPIP(5)K:dSopE, −320%). The phenotypic similarity between tPIP(5)K:dSopB and tSipC:dSopB suggested that SipC might generate PI(4,5)P_2_ either directly or via PIP(5)K stimulation. Indeed, the periphery of *Salmonella*-induced membrane ruffles are enriched for PI(4,5)P_2_ [20].

To pursue this hypothesis, we assessed the relative proportion of phosphatidylinositol species in cells expressing *Salmonella* effectors by radioactive counting of fractions separated by ion exchange chromatography (**Figure 8B**). When compared to resting cells, tSopB lysates contained significantly enhanced levels of phosphatidylinositol-3,4,5-trisphosphate [PI(3,4,5)P_3_] and phosphatidylinositol-3,4-bisphosphate [PI(3,4)P_2_], likely to be a stable breakdown product of PI(3,4,5)P_3_ [27], and to a lesser extent phosphatidylinositol-3-phosphate [PI(3)P] (**Figure 8B, tSopB**). By comparison, no major alterations were observed when comparable tSipC or tSptP lysates were assayed (**Figure 8B, tSipC** and **tSptP**). These biochemical data demonstrate that SipC itself does not directly or indirectly generate PI(4,5)P_2_, but do not preclude that SipC alters the local concentration or distribution of existing PI(4,5)P_2_ in the membrane. To examine this possibility, the PI(4,5)P_2_-binding plextrin-homology (PH) domain of phospholipase Cδ fused to green fluorescent protein (PLCδ-PH-GFP) was expressed in cultured cells prior to infection with effector-augmented strains to report PI(4,5)P_2_ distribution during invasion [20]. As expected, the membrane ruffles associated with actin rearrangements induced by the dSopB strain were largely devoid of PI(4,5)P_2_, indicated by the diffuse reporter probe distribution (**Figure 8C**, dSopB PLCδ-PH-GFP). In contrast, membrane ruffles generated by the dSipC strain were enriched with PLCδ-PH-GFP, which was frequently coincident with induced actin rearrangements and concentrated at filopodial tips (**Figure 8C**, dSipC). Clusters of PLCδ-PH-GFP were also clearly evident at the plasma membrane, a distribution strikingly similar to that of SipC itself [8],[19]. Although cells infected with the dSopE strain also induced profuse membrane ruffles morphologically more reminiscent of the dSipC strain [19], no PI(4,5)P_2_ enrichment or clustering was evident (**Figure 8C**, dSopE). These data suggest that localized accumulation or clustering of plasma membrane PI(4,5)P_2_ by SipC enhances the availability of SopB substrate at bacterial entry foci. These findings from biochemical and cell biology approaches demonstrate the physiological context of a novel relationship highlighted by our genetic BENEFIT screening and extend our mechanistic understanding of SipC-SopB interplay.

## Discussion

**Figure 9:**
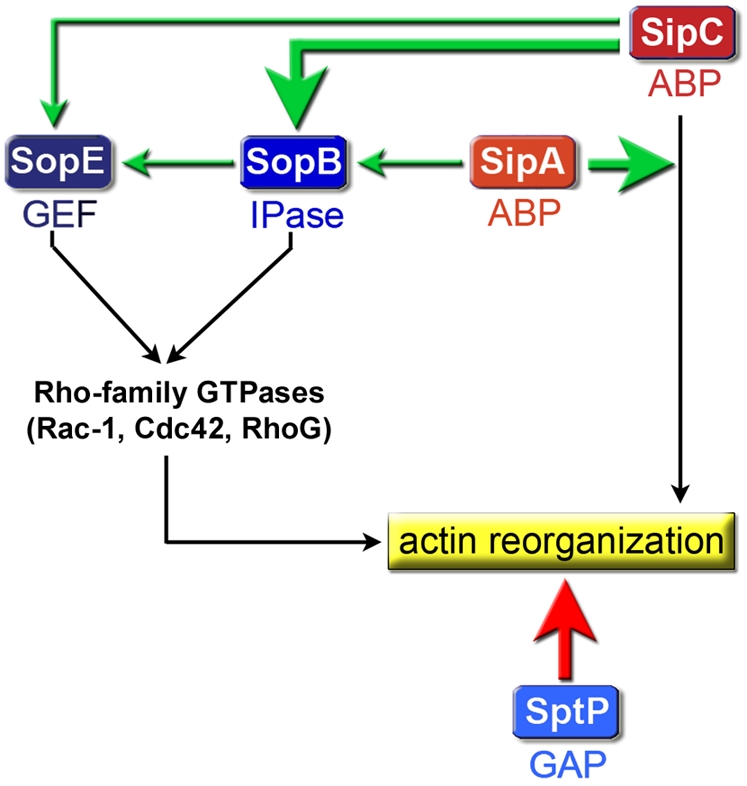
Interplay between delivered *Salmonella* entry effectors. Schematic summary of the synergistic (green arrows) and antagonistic (red arrow) relationships between delivered *Salmonella* invasion effectors revealed by BENEFIT screening. Arrow width depicts relative magnitude of functional cooperativity. Abbreviations, ABP (actin-binding protein), GEF (GTPase exchange factor), IPase (inositol phosphatase), GAP (GTPase activating protein).

Emerging evidence has inferred that multiple effectors delivered by bacterial T3SSs act cooperatively within the target cell to subvert host signaling processes [8],[17],[28]. *Salmonella* invasion of host cells is a paradigm system in which the biochemical activities of the central T3SS effectors that manipulate cellular actin dynamics are likely identified [6],[11]. However, the nature and extent of cross talk between these effectors that co-localize at the target cell plasma membrane remained unknown [19]. Our findings provide initial insights into a sophisticated program of ordered effector activities underlying cell invasion by *Salmonella* **(** **Figure 9** **)**. The BENEFIT screening data identify extensive novel Sip-Sop synergy (SipC-SopB; SopB-SipA; SipC-SopE), and additionally validate every cooperative [SipA-SipC [8]; SopB-SopE [16]] and antagonistic [SopE-SptP [17]] effector relationship detected by previous genetic or biochemical approaches.

We demonstrate that SipC promotes actin polymerization independently of Rho-family GTPases. This is clearly consistent with the ability of SipC to nucleate actin polymerization directly [7], and support a fundamental role for this function during invasion [22],[29]. SipC also emerges as a central participant in a more extensive signaling network, as it engages in potent synergistic relationships with delivered SipA and SopB, and to a lesser extent with SopE. Such early intracellular communication between SipC and Sops reconciles the observation that triple *Salmonella* mutants lacking *sopE*, *sopE2* and *sopB* are unable to efficiently induce actin rearrangements [16]. Network architecture implies that SipC not only cooperates with SipA to directly trigger actin reorganization [8], but also concurrently reinforces signaling by indirectly stimulating SopB and SopE activity. This ‘feed-forward’ amplification and evolved effector interdependency may allow the Cdc42/Rac1-dependent pathway to dominate during WT infection [24].

Our data reveal an important role for phosphoinositides in mediating the dominant and previously unrecognized synergy between SipC and SopB. PI(4,5)P_2_ clusters in membrane ruffles generated specifically by SipC, and is subsequently hydrolysed by SopB. As only the dSopB strain gains from co-infection with the dSipC strain, this suggests that prior PI(4,5)P_2_ concentration at entry sites either by dSipC or experimentally with tSipC subsequently enhances the invasion of the dSopB strain, which is more adept at ruffle closure by virtue of augmented inositol phosphatase activity. Like SipC, we show that PI(4,5)P_2_ concentrates locally at the tip of induced filopodia [19]. This is consistent with co-secreted cholesterol-binding SipB initially integrating into cholesterol-rich regions of the plasma membrane [5], where PI(4,5)P_2_ also concentrates. It is also tempting to speculate that PI(4,5)P_2_ might also influence bacterial SipC actin nucleation activity, as this species is a stimulatory co-factor of cellular Arp2/3-dependent actin polymerization [30],[31]. Similarly, our findings that tSopB generates PI(3,4,5)P_3_, PI(3,4)P_2_ and PI(3)P concurs with reports of SopB-dependent stimulation of RhoG via SGEF and PI(3)P accumulation on *Salmonella*-containing vacuoles [12],[32].

BENEFIT screening not only defines such previously undetected relationships between delivered effectors, but also supports the view that a controlled programme of temporal effector activities drives *Salmonella* invasion. In the *trans* screen, the detected synergies are unidirectional, *i.e.* tSipC enhances invasion of the dSopB strain but not *vice versa*, an effect recapitulated in the *cis* screen, when co-infecting strains synergise selfishly or mutually. Also, tSopB or tSptP expression in cells prior to infection selectively inhibits invasion of particular augmented strains. This experimentally imposed asynchronous SopB or SptP activity likely inhibits subsequently delivered effectors by sequestering or down-regulating essential cellular targets (Rho family GTPases) or by prematurely inter-converting necessary secondary messengers through SopB inositol phosphatase activity or SptP-dependent protein dephosphorylation. As tSptP also reduces WT invasion, this might reflect a premature generalized GAP-driven protective effect [17]. However, tSopB only inhibits the invasion of certain effector-augmented strains (*i.e*. not the WT) indicative of specific functional interplay, a phenotype abolished when tSopB derivatives are expressed that lack inositol phosphatase activity. The BENEFIT screens do not distinguish whether these temporal relationships reflect the ordered activity of simultaneously delivered effectors or a requirement for sequential effector delivery via the T3SS. The available experimental data from WT infection still remain consistent with either scenario: delivered effectors co-localize at the plasma membrane [19]; ‘early’ acting SopE and ‘late’ SptP are present in cells at equimolar concentrations after 15 minutes [33]; and imaging of effector delivery suggested equivalent kinetics for both SipA and SopE transfer [34]. What is clear from our data is that the T3SS has evolved to ensure that delivered effectors are precisely dosed in the WT to balance efficient bacterial invasion and host cell viability.

Although most effector relationships are common to both BENEFIT screens, SipA-SopB synergy that favoured the dSopB strain emerged uniquely from the *cis* screen. Why this phenotype was constrained to mixed infection is as yet unclear, although it might relate to the recently identified role of these effectors in invasion-associated tight junction disruption [35]. Provocatively, both these effectors also have additional intracellular roles in controlling the subsequent maturation and positioning of *Salmonella* containing vacuoles [32],[36], although whether these functions are simultaneously activated 60 minutes after infection remains unknown.

Our data provide the first systematic experimental study examining the scope, nature and relative potency of interplay between bacterial effectors delivered into eukaryotic cells. Nevertheless, as with any experimental system BENEFIT screens inevitably also have associated limitations. These include the use of a cultured cell model and genetically modified *Salmonella* strains, and the possibility that changes in invasion rate used as a readout result from temporal alterations or asynchrony in the process or differential effector stability rather than interplay. These aspects are technically challenging to monitor and control. However, coupled to the fact that our screening detected every previously identified effector association, our extended investigation of the novel SipC-SopB synergy using complementary biochemical and cell biology approaches, provides strong supporting evidence that the previously unrecognized relationships emerging from our screening reflect *bona fide* effects. Nevertheless, each of the identified associations now requires further in depth investigation.

Biochemical and structural studies have illustrated that many effectors comprise defined domains that act as functional modules [37], for example SipA contains a C-terminal actin-binding domain and an N-terminal region that influences phagosome positioning [9],[36]. As we have demonstrated with our analysis of SipC-C and SipC-N function and the catalytic-dead SopB derivative, BENEFIT screening assays can now be exploited further to decipher the relative contribution of such discrete functions to effector interplay, and to examine how as yet undefined *Salmonella* effectors might contribute to invasion. As demonstrated using Rho-family GTPases as proof-of-principle, dominant negative and RNAi approaches can also be employed to examine the involvement of additional host targets in distinct effector-stimulated signaling pathways. The assays could also be adapted to examine virulence proteins in other pathogen-induced processes, provided that there is a readily quantifiable output phenotype such as bacterial replication or dissemination.

## Materials and Methods

### Bacterial strains and cell culture

Construction of *S.typhimurium* SL1344 strains engineered to express augmented levels of individual entry effectors in a WT background has been described in detail previously [19]. Briefly, *S.typhimurium* SL1344 was transformed with expression plasmids pB:SopE_FLAG_, pB:SopB_FLAG_, pB:SopB_FLAG_ pB:SipA, pB:SptP_FLAG_, pB:SipC, pB:SipB or pB:SipD generated by PCR amplification of *sopE*, *sopB*, *sipA*, *sptP*, *sipC, sipB* or *sipD* engineered to contain *Nde*I and *Hind*III sites from an SL1344 chromosomal DNA template, into pTrc99A-FF4 to allow constitutive low-level expression downstream of the Trc promoter [38],[39]. pB:SopB^C462S^_FLAG_ was generated identically, except the TGT codon at nucleotide 1383 of *sopB* was mutated to TCC to generate a cysteine to serine substitution at amino acid 462. Immunoblotting and densitometry of cell lysates and secreted proteins from each strain showed that plasmid-encoded SopE, SopB, SipA, SptP or SipC expression was specifically but mildly augmented (1.5–3.3±0.28 fold WT) due to the low copy number plasmid in the absence of induction, and that this elevated expression correlated with increased effector secretion (1.5–2.0±0.28 fold WT). This also correlated with a mild increase in concentrations of delivered effectors, as each could be detected by immunofluorescence or immunoblotting of cell lysates after infection with the engineered strains but were below the detection threshold after WT infection under the same conditions [19]. Exogenous expression of plasmid-encoded effectors did not interfere with the expression, secretion or delivery of other chromosomal SPI1- or SPI2-secreted effectors [[19], and L.Brawn, unpublished observations]. Expression and secretion of SipB and SipD are documented in **Figure S1**. *S.typhimurium* SL1344 strains were maintained on Luria-Bertani agar or cultured in tryptone yeast medium (2TY) containing ampicillin or spectinomycin (50 µgml^−1^), as appropriate.

NIH3T3 fibroblasts were cultured in Dulbecco's modified Eagle's medium (DMEM) supplemented with 10% (v/v) fetal calf serum (FCS), L-glutamine and penicillin/streptomycin (Sigma/Invitrogen) (37°C, 5% CO_2_). When required cells were treated with bradykinin (30 ngml^−1^; 37°C, 5% CO_2_, 60 min). To assess number and viability, ∼1×10^6^ cells were scraped into PBS, ice cold 70% (v/v) ethanol added (10vol), and the mixture incubated (4°C, 30 min). Cells were then pelleted (900g, 5 min) and resuspended in PBS. RNAse and propidium iodide were added, and the mixture incubated (37°C, 30 min). Cell clumps were dispersed by passage through a 25-gauge needle and samples analysed for forward scatter, side scatter and fluorescence on a FACScan flow cytometer (Becton Dickinson, USA), collecting >20000 events per sample.

Eukaryotic expression vectors pT:SopE_FLAG_, pT:SopB_FLAG_ and pT:SptP_FLAG_ containing *sopE*, *sopB*, and *sptP* were generated by PCR amplification from the *S.typhimurium* SL1344 chromosome and cloning of products, engineered to contain *Bam*HI and *Xho*I sites into pT:_FLAG_ [19], a fusion of *Alw*NI-digested phrGFP-NUC and pIRES-hrGFP (Stratagene). pT:SipA, pT:SipC and pT:SipB were constructed by cloning of *sipA*, *sipC* or *sipB* PCR products engineered to contain *Xba*I and *Hind*III sites into pcDNA3.1 (Invitrogen). pT:SipCN_FLAG_ and pT:SipCC_FLAG_ were generated by PCR amplification of nucleotides 1–360 and 600–1230 of *sipC* corresponding to amino acids 1–120 and 200–409 and cloning into pT:_FLAG_. pT:IpgD_FLAG_ was generated by PCR amplification of *ipgD* from the *Shigella flexneri* M90T virulence plasmid and cloning into pT:_FLAG_. pT:SopB^C462S^_FLAG_ was generated as described for pB:SopB^C462S^_FLAG_, except that the PCR product was cloned into pT:_FLAG_. pPLCδ-PH-GFP expressing the PH-domain of phospholipase Cδ that specifically binds phosphatidylinositol-4,5-bisphosphate [PI(4,5)P_2_] fused to C-terminal GFP was a kind gift from Phill Hawkins (Babraham Institute, Cambridge). pT:*PIP(5)K* was generated by PCR amplification of the cDNA encoding murine phosphatidylinositol-4-phosphate-5-kinase and cloning of the product into pcDNA3.1.

### BENEFIT screens

The ‘*cis’* or ‘*trans’* binary entry effector interplay (BENEFIT) screens are a modification of the gentamicin protection assay, which measures the ability of *Salmonella* strains to invade cultured cells [40].

### ‘*trans*’ BENEFIT screen

Stationary phase *S.typhimurium* SL1344 cultures transformed with pB:*sopE*_FLAG_, pB:*sopB*_FLAG_, pB:*sptP*_FLAG_, pB:*sipC*_FLAG_, pB:*sipA*, pB:*sipB*, pB:*sipD* or vector control [19], were diluted 1∶500 in 2TY and incubated to maximise invasion efficiency (6 h, 37°C, 225 r.p.m.). Bacteria were washed and resuspended in DMEM supplemented with L-glutamine, and added at a multiplicity of infection (MOI) of 50 to 2×10^4^ serum-starved NIH3T3 fibroblasts, transfected 48 h previously with expression plasmids pT:*sopE*_FLAG_, pT:*sopB*_FLAG_, pT:*sopB^C462S^*_FLAG_, pT:*sptP*_FLAG_, pT:*sipC*_FLAG_, pT:*sipCN*_FLAG_, pT:*sipCC*_FLAG,_ pT:*sipA*, pT:*ipgD*_FLAG_, pT:*PIP(5)K* or empty vector [19] using Lipofectamine™, according to the manufacturer's instructions (Invitrogen). Transfection efficiency, assessed by GFP-NLS co-expression or immunofluorescence [19] was always >85%. After incubation (37°C, 5% CO_2_, 60 min), cells were repeatedly washed with warm PBS and extracellular bacteria killed with gentamicin (100 µgml^−1^ in DMEM; 37°C, 5% CO_2_, 60 min). Cells were washed again with PBS and lysed in 10 mM Tris-Cl, pH 7.4, 0.5% (v/v) triton X-100. Serial lysate dilutions were plated onto LB agar and the percentage of intracellular bacteria compared to the original inoculum. Rates were referenced against invasion of WT *S.typhimurium* SL1344 and an isogenic invasion deficient mutant lacking *invG*, an essential structural component of the T3SS, assigned as 100% and 0% respectively, in each transfectant background. Functional interplay between effector A (‘d*effector*’; delivered via the T3SS) and effector B (‘t*effector*’ pre-expressed in the target cell by transient transfection) was defined as an increase (or decrease) in rate of at least one-fold (*i.e.* ±100%) of WT after correction for WT *Salmonella* invasion of cells expressing effector B and invasion of control cells by WT *Salmonella* expressing augmented levels of effector A.

### ‘*cis*’ BENEFIT screen

Stationary phase *S.typhimurium* SL1344 cultures transformed with pB:*sopE*_FLAG_, pB:*sopB*_FLAG_, pB:*sptP*_FLAG_, pB:*sipC*_FLAG_, pB:*sipA* or empty vector [19], were diluted 1∶500 in 2TY and incubated to maximise invasion efficiency (6 h, 37°C, 225 r.p.m.). Bacteria were washed and resuspended in DMEM supplemented with L-glutamine and mixed at a 50∶50 or 90∶10/10∶90 ratio as appropriate to give an MOI of 50, when 2×10^4^ serum-starved NIH3T3 fibroblasts were infected. An antibiotic marker (spectinomycin) was introduced into one strain to allow the relative invasion efficiency of each strain to subsequently be calculated. Since selection conferred a marginal disadvantage (∼5%), each infection was performed in duplicate to allow the marker to be alternated. After incubation (37°C, 5% CO_2_, 60 min), cells were repeatedly washed with warm PBS and extracellular bacteria killed with gentamicin (100 µgml^−1^ in DMEM; 37°C, 5% CO_2_, 60 min). Cells were washed again with PBS and lysed in 10 mM Tris-Cl pH 7.4, 0.5% (v/v) triton X-100. Serial dilutions were plated onto LB agar with and without spectinomycin and the percentage of intracellular bacteria compared to the original inoculum. In the *cis* assay, functional interplay between effector A (‘dA’; delivered via the T3SS) and effector B (‘dB’; delivered by the T3SS of a separate strain) was defined as an increase (or decrease) in rate of at least one-fold (*i.e.* ±100%) of WT after correction for the rate expected when either strain invades cells independently.

In both screens, the results presented are the mean±SEM of 4 independent experiments each performed in triplicate and the changes reported statistically significant from the controls (Mann Whitney U p<0.05).

### Immunofluorescence microscopy

Infected or transfected cells were washed in PBS and fixed with 3.7% (v/v) paraformaldehyde for fluorescence microscopy. Fixed samples were permeabilized with 0.2% (v/v) triton X-100 in PBS, blocked with 3% (w/v) bovine serum albumin (BSA) in PBS (1 h, RT), then incubated with appropriately diluted primary antibodies (anti-FLAG monoclonal or anti-effector polyclonal) in PBS (1h, RT). Samples were sequentially incubated with AlexaFluor 488-conjugated anti-rabbit or anti-mouse IgG secondary antibodies, according to manufacturer's instructions (30 min, RT; Invitrogen), then Texas Red-conjugated phalloidin (20 min, RT; Invitrogen) and 4′,6′-diamidino-2-phenylindole dihydrochloride (DAPI, Sigma) in PBS. Coverslips were mounted using ProLong Anti-fade reagent (Invitrogen), and visualized using a fluorescence microscope (Leica DM IRBE). Images were captured using a CCD digital camera (Hamamatsu) and processed using OpenLab software (Improvision). Where appropriate GFP fluorescence was observed directly in fixed cells. Bacterial DAPI signal was artificially enhanced to normalise the intensity to that of nuclear staining.

### Determination of effector concentration

Effector protein concentrations in transfected cells or delivered into cells (measured 60 min after *S.typhimurium* infection) were assayed by immunoblotting mechanical cell lysates or subcellular fractions with antibodies raised against purified effectors [19]. Signal intensities were determined using NIH Image freeware (http://rsb.info.nih.gov/nih-image/), and intensity values compared to those derived by immunoblotting known concentrations of purified effectors. Cells were fractionated as previously described [19].

### Quantitative analysis of inositol phospholipids in cultured cells

Analysis was carried out as described [41]. Briefly, transfected NIH3T3 cells were washed and incubated with phosphate-free DMEM supplemented with 2% (v/v) dialysed heat-inactivated FCS, 0.2% (w/v) fatty acid-free BSA and ∼0.3 mCi [^32^P]P_i_ ml^−1^ in 10 mM HEPES pH7.4 (diluted from an iso-osmotic stock) to allow labelling of cellular phosphoinositides (37°C, 5% CO_2_, 75 min). Cells were rapidly aspirated and fixed with 1 vol ice-cold 1.0 M HCl, and scraped into glass vials containing 4 vol CHCl_3_:MeOH (2∶1, v/v), Folch carrier lipid and tetrabutylammonium sulphate (to give final ratio aqueous/MeOH/CHCl_3_ 3∶4∶8). This was vortexed and centrifuged (1000g, 5 min) to separate phases. The lower phase was removed into fresh tubes containing synthetic upper phase, mixed and centrifuged (1000 g, 5 min), then dried *in vacuo*. Dried lipids were deacylated by addition of monomethylamine reagent [41], warmed (53°C, 5 min), vortexed and incubated (53°C, 25 min). Samples were subsequently cooled (to RT) and dried *in vacuo*. Deionized H_2_O and petroleum ether/n-butanol/ethyl formate [4∶20∶1 (v/v)] were then added, then the mixture vortexed and centrifuged (1000 g, 5 min). The upper organic phase was then removed, the lower water-soluble phase mixed with 1 vol petroleum ether/n-butanol/ethyl formate [4∶20∶1 (v/v)], and the mixture vortexed and centrifuged as previously. The lower phase and interface was then dried *in vacuo*. Labelled lipid species were then separated for analysis by high-performance liquid chromatography (HPLC). Dried lipids were resuspended in dH_2_O (2 ml; bath sonicated, and vortexed) and filtered (0.45 µm). Samples were loaded onto a pre-equilibrated Whatman Partisphere 5SAX column (12.5 cm) and developed at 1 mlmin^−1^ using a gradient of H_2_O (A) versus 1.25 M NaH_2_PO_4_ (B) [0 min, 0% B; 1 min, 1% B; 30 min, 6% B; 31 min, 15% B; 60 min, 25% B; 61 min, 33% B; 80 min, 60% B; 81 min, 100% B]. Fractions were collected every 30 s and scintillant added (Packard ‘Ultima Gold’). Lipid species were identified from the elution profile by comparison to labelled standards of known individual phosphoinositide species.

PI(5)P levels were assayed as described [42]. Briefly, cultured cells were lysed into ice cold 5% (v/v) perchloric acid and incubated on ice prior to centrifugation (5000g, 10 min, 4°C). Lipids were extracted from the pellet using acidified chloroform/methanol and an aliquot removed for phospholipid quantification such that PI_5_P levels could be assessed relative to total cellular lipid [42]. The remainder was dried and resuspended in 1 vol CHCl_3_∶CH_3_OH∶ammonium formate [5∶10∶2 (v/v/v), final concentration 50 mM ammonium formate). This was added to 0.1 vol neomycin beads [42] successively washed in 1 vol CHCl_3_∶CH_3_OH∶H_2_O [5∶10∶2 (v/v/v)], CHCl_3_:CH_3_OH:ammonium formate [5∶10∶2 (v/v/v), final concentration 0.5 M formate], and finally resuspended in 0.1 vol CHCl_3_:MeOH:ammonium formate [5∶10∶2 (v/v/v), final concentration 50 mM formate]. Samples were incubated (RT, 20 min), centrifuged (4000 g, 1min) and the supernatant discarded. Beads were washed twice with 1 vol 50 mM ammonium formate buffer and lipids eluted twice with 2 M triethylbicarbonate (TEAB) to which phosphatidylserine was added as an inert carrier (14.25 µM). Beads were incubated with 0.5 vol 2 M TEAB and phosphatidylserine (1 h), centrifuged (4000 g, 2 min) and supernatants collected. Beads were washed again with 0.2 vol 2 M TEAB and phosphatidylserine (1 h), and supernatants combined with the first. Tubes were dried *in vacuo* (60°C, overnight). Lipid phosphorylation with recombinant type IIα phosphatidylinositol kinase to determine PI(5)P mass was performed as previously [42]. Phosphorylation was initiated by addition of 2 µCi [γ-^32^P] ATP and 5 µM ATP to each sample, and PI(4,5)P_2_ extracted and separated by thin layer chromatography. PI(5)P levels were determined from a standard curved with reference to known concentrations of synthetic PI(5)P, and expressed relative to amount of total phospholipids present in each sample.

## Supporting Information

Figure S1 Characterization of *S.typhimurium* strains and cultured cells exogenously expressing SipB and SipD. A. Effector expression and secretion in *S.typhimurium* SL1344 wild-type compared with strains transformed with pTrc plasmids expressing SipB or SipD *in trans* in the wild-type background. Equivalent loadings of late exponential cell lysates (left) and filtered culture supernatants (right) were separated by SDS-PAGE and analyzed by immunoblotting with the appropriate effector antibody or RfaH (control). Densitometric analysis showed that plasmid-encoded SipB and SipD expression was specifically augmented in the absence of induction (∼4-fold wild-type), and that elevated expression correlated with increased effector secretion (∼3-fold wild-type). Effector expression from the plasmid did not interfere with the expression or secretion of other chromosomally-encoded entry effectors. B. Effect of exogenous SipB or SipD expression on *S.typhimurium* entry into cultured fibroblasts. Results of gentamicin protection assays using wild-type or strains exogenously expressing SipB (dSipB) or SipD (dSipD). Entry rates (after 60 min) were compared to wild-type (assigned as 100%) containing the empty plasmid. Results are the mean of four independent experiments each with three replications. Correspondingly, no significant change was observed in mean cells invaded per field (wild-type 5±0.5, dSipB 5±1, dSipD 5±1; numbers of invaded cells were scored in >30 cells in each of three independent experiments), or in mean number of bacteria per fibroblast (wild-type 1.65±0.1, dSipB 1.48±0.23, dSipD 1.0±0.28; bacteria were scored in >100 cells by inside/outside immunofluorescence staining in each of three independent experiments. C. Immunofluorescence localization of SipB and SipD in *Salmonella*-infected fibroblasts. Immunofluorescence micrographs of fixed NIH3T3 cells after infection (60 min) with *Salmonella* strains expressing augmented levels of SipB or SipD as indicated. Left column shows triple fluorescence (merge) of cells stained with anti-SipB or anti-SipD/Alexafluor 488-conjugated anti-rabbit IgG to localize each effector (green), Texas Red-conjugated phalloidin to visualize F-actin (red), and DAPI to localize cell nuclei and bacteria (blue). Middle column shows individual effector channel (effector) and right column the DAPI channel from which the nuclear signal has been removed for clarity (indicated by dashed lines) (bacteria). Arrows indicate internalized bacteria, and areas indicated in merge are show at higher magnification in inserts. Scale bars, 2 µm. D. Immunofluorescence micrograph of fixed NIH3T3 fibroblast transiently expressing SipB. Left panel shows double fluorescence (merge) image of cell SipB stained 48 h after transfection with anti-SipB polyclonal antiserum/Alexafluor 488-conjugated anti-rabbit IgG to localize SipB (green), and Texas Red-conjugated phalloidin to visualize F-actin (red). SipB channel alone (effector) is shown in greyscale for clarity. Image is typical of >50 cells from three independent experiments. Scale bar, 2 µm. E. Localization of SipB and SipD in *Salmonella*-infected and effector-transfected fibroblasts. Cultured NIH3T3 fibroblasts were infected (60 min) with S.typhimurium strains expressing augmented levels of SipB (dSipB) or SipD (dSipD) in the wild-type background or were transiently transfected (48 h) with an expression vector encoding *S.typhimurium* SipB. Cells were mechanically fractionated (as [19]), and each subfraction [nuclei+internalized bacteria (N+B), cytoskeleton and internal membranes (CS/IM), cytoplasm (C), plasma membrane (PM)] separated by SDS-PAGE and analysed by immunoblotting with anti-SipB or anti-SipD antibody. SipD was below the detection threshold under these conditions.
(13.32 MB TIF)

Figure S2 Intracellular effector concentration after T3SS-dependent delivery and transfection. A. Cell number and viability following transfection with *Salmonella* effectors. NIH3T3 fibroblasts were individually transfected with indicated effectors and analyzed by flow cytometry after 48 h. Cell number is expressed as the percentage of control cells, with apoptotic fraction shaded black. B. Effector concentration following delivery or transfection. Upper: Cells transfected with effectors (t-effector) or infected with effector-augmented (d-effector) *S.typhimurium* strains were mechanically fractionated prior to immunoblotting with appropriate anti-effector antibodies. Effector concentration in the internal membrane/cytoskeletal (IM/CS) and plasma membrane (PM) fractions was determined by densitometric analysis of band intensity and comparison to purified protein standards (*e.g.* SipC and SopE). To facilitate simultaneous quantification of effectors after transfection and infection, fractions from 2.7×10^6^ and 5.4×10^6^ cells were immunoblotted, respectively. Lower: Graph shows comparative concentrations of each transfected and delivered effector. Split bars represent relative concentration in the two subcellular fractions (IM/CS lower division; PM upper division). C. Effector localization following delivery and transfection. Cells transfected with effectors (*e.g.* tSipC, tSipA, tSopE or tSptP) were infected with effector-augmented (d-effector) *S.typhimurium* strains (*e.g.* dSipA, dSipC, dSptP or dSopE). Infected transfectants were mechanically fractionated, each sub-fraction [N+B cell nuclei+internalized bacteria; IM/CS internal membranes/cytoskeleton; C cytoplasmic; PM plasma membrane] separated by SDS-PAGE, and analyzed by immunoblotting with the appropriate anti-effector antibody.
(0.53 MB TIF)

Figure S3 The T3SS translocator SipB and SipD are not invasion effectors. A. Schematic illustrating *trans* BENEFIT screening (the infection of cells expressing individual entry effectors by wild-type WT or effector-augmented *S.typhimurium* strains). Wild-type (WT) bacteria endogenously express, secrete and deliver *sipA, sipC, sopE, sopB* and *sptP* (abbreviated to *acebp*) and *sipB* and *sipD* (abbreviated separately as *bd*). Effector-augmented strains each express, secrete and deliver mildly increased levels of an individual plasmid-encoded effector in the WT background [enhanced effector shown in capitals, *e.g. acebp Bd* (dSipB) and *acebp bD* (dSipD) produce increased levels of SipB and SipD, respectively]. Cultured cells were transfected with individual entry effectors (denoted t-effector) prior to infection. B. Upper: Cultured fibroblasts were transfected (t) with individual effectors prior to infection with WT or effector-augmented (d-effector) *S.typhimurium* strains. Invasion rates after 60 min were compared to WT (assigned as 100%). Results are mean±SEM of 4 independent experiments each performed in triplicate. Baselines ‘wild-type’ and ‘Δ*invG*’ denote *S.typhimurium* SL1344 and *S.typhimurium* Δ*invG* (T3SS deficient) invasion in each transfectant background, respectively. Lower: Table showing differences in invasion rates (%) after correction. Shading denotes a significant increase (green), significant decrease (red) or no significant change (grey) in invasion (Mann Whitney U p<0.05).
(9.10 MB TIF)

Figure S4 Actin rearrangements induced during infection of transfected cells. Cultured fibroblasts were transfected with indicated effectors (t-effector) and subsequently infected with effector-augmented (d-effector) *S.typhimurium* strains. Cells were fixed 60 min post infection and double fluorescence stained to visualise F-actin and bacteria/cell nuclei. Arrows indicate internalized bacteria.
(1.66 MB TIF)

Figure S5 Effector synergy is abolished by biasing the relative levels of each strain. *S.typhimurium* SL1344 or effector augmented (d-effector) strains were mixed pair wise (MOI 50) at 50∶50, 90:10 and 10:90 ratios. Invasion of each strain was assessed using selectable markers after 60 min (Figure 4A). Results are the mean of four independent experiments each performed in triplicate. Pie charts depict total invasion by each combination (size; combined %) and relative contribution of each strain (division; %) using the indicated ratios.
(0.69 MB TIF)
