# Supplementary figures and images for: Deciphering Interplay between Salmonella Invasion Effectors
Source: PLoS Pathog. 2008 Apr 4;4(4):e1000037. doi: 10.1371/journal.ppat.1000037 (PMC2268969; doi:10.1371/journal.ppat.1000037)

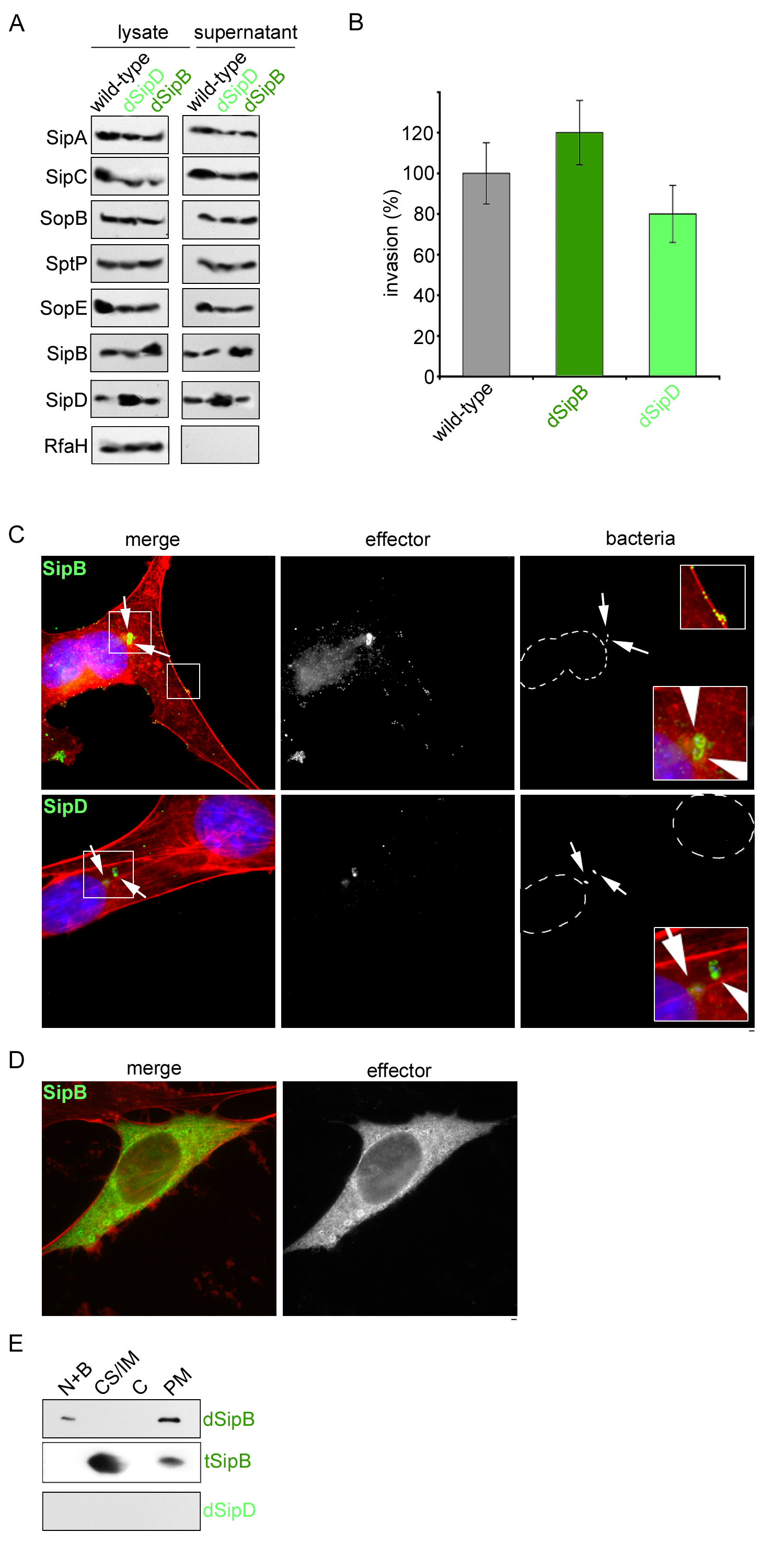

Supplement: Figure S1 — Characterization of S.typhimurium strains and cultured cells exogenously expressing SipB and SipD. A. Effector expression and secretion in S.typhimurium SL1344 wild-type compared with strains transformed with pTrc plasmids expressing SipB or SipD in trans in the wild-type background. Equivalent loadings of late exponential cell lysates (left) and filtered culture supernatants (right) were separated by SDS-PAGE and analyzed by immunoblotting with the appropriate effector antibody or RfaH (control). Densitometric analysis showed that plasmid-encoded SipB and SipD expression was specifically augmented in the absence of induction (∼4-fold wild-type), and that elevated expression correlated with increased effector secretion (∼3-fold wild-type). Effector expression from the plasmid did not interfere with the expression or secretion of other chromosomally-encoded entry effectors. B. Effect of exogenous SipB or SipD expression on S.typhimurium entry into cultured fibroblasts. Results of gentamicin protection assays using wild-type or strains exogenously expressing SipB (dSipB) or SipD (dSipD). Entry rates (after 60 min) were compared to wild-type (assigned as 100%) containing the empty plasmid. Results are the mean of four independent experiments each with three replications. Correspondingly, no significant change was observed in mean cells invaded per field (wild-type 5±0.5, dSipB 5±1, dSipD 5±1; numbers of invaded cells were scored in >30 cells in each of three independent experiments), or in mean number of bacteria per fibroblast (wild-type 1.65±0.1, dSipB 1.48±0.23, dSipD 1.0±0.28; bacteria were scored in >100 cells by inside/outside immunofluorescence staining in each of three independent experiments. C. Immunofluorescence localization of SipB and SipD in Salmonella-infected fibroblasts. Immunofluorescence micrographs of fixed NIH3T3 cells after infection (60 min) with Salmonella strains expressing augmented levels of SipB or SipD as indicated. Left column shows triple [file ppat.1000037.s001.tif]

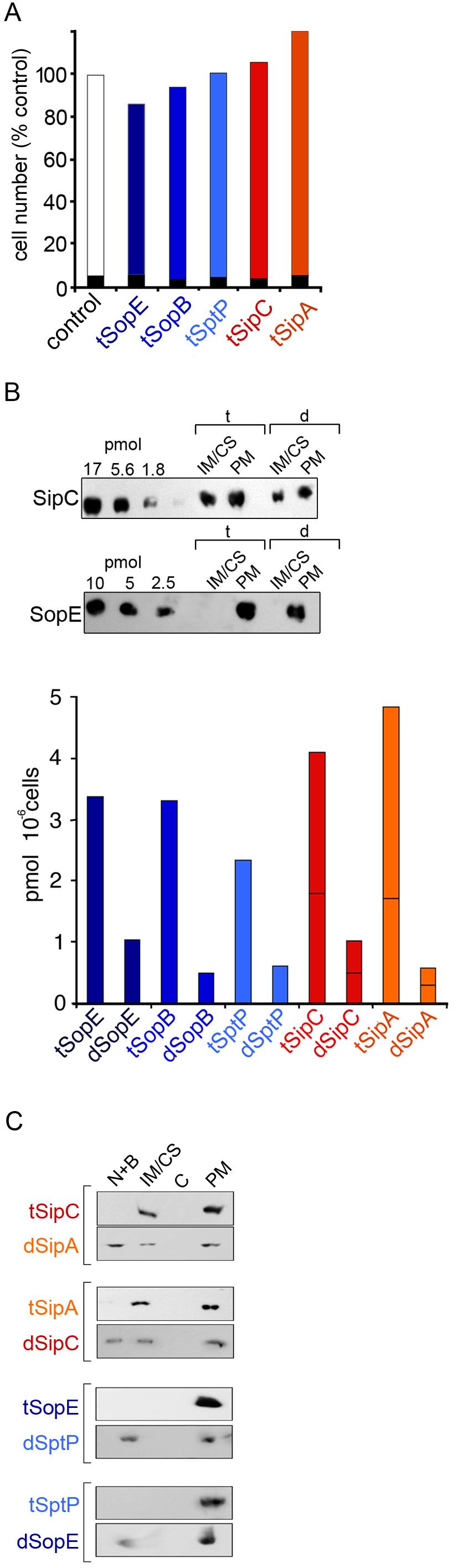

Supplement: Figure S2 — Intracellular effector concentration after T3SS-dependent delivery and transfection. A. Cell number and viability following transfection with Salmonella effectors. NIH3T3 fibroblasts were individually transfected with indicated effectors and analyzed by flow cytometry after 48 h. Cell number is expressed as the percentage of control cells, with apoptotic fraction shaded black. B. Effector concentration following delivery or transfection. Upper: Cells transfected with effectors (t-effector) or infected with effector-augmented (d-effector) S.typhimurium strains were mechanically fractionated prior to immunoblotting with appropriate anti-effector antibodies. Effector concentration in the internal membrane/cytoskeletal (IM/CS) and plasma membrane (PM) fractions was determined by densitometric analysis of band intensity and comparison to purified protein standards (e.g. SipC and SopE). To facilitate simultaneous quantification of effectors after transfection and infection, fractions from 2.7×10^6^ and 5.4×10^6^ cells were immunoblotted, respectively. Lower: Graph shows comparative concentrations of each transfected and delivered effector. Split bars represent relative concentration in the two subcellular fractions (IM/CS lower division; PM upper division). C. Effector localization following delivery and transfection. Cells transfected with effectors (e.g. tSipC, tSipA, tSopE or tSptP) were infected with effector-augmented (d-effector) S.typhimurium strains (e.g. dSipA, dSipC, dSptP or dSopE). Infected transfectants were mechanically fractionated, each sub-fraction [N+B cell nuclei+internalized bacteria; IM/CS internal membranes/cytoskeleton; C cytoplasmic; PM plasma membrane] separated by SDS-PAGE, and analyzed by immunoblotting with the appropriate anti-effector antibody. (0.53 MB TIF) [file ppat.1000037.s002.tif]

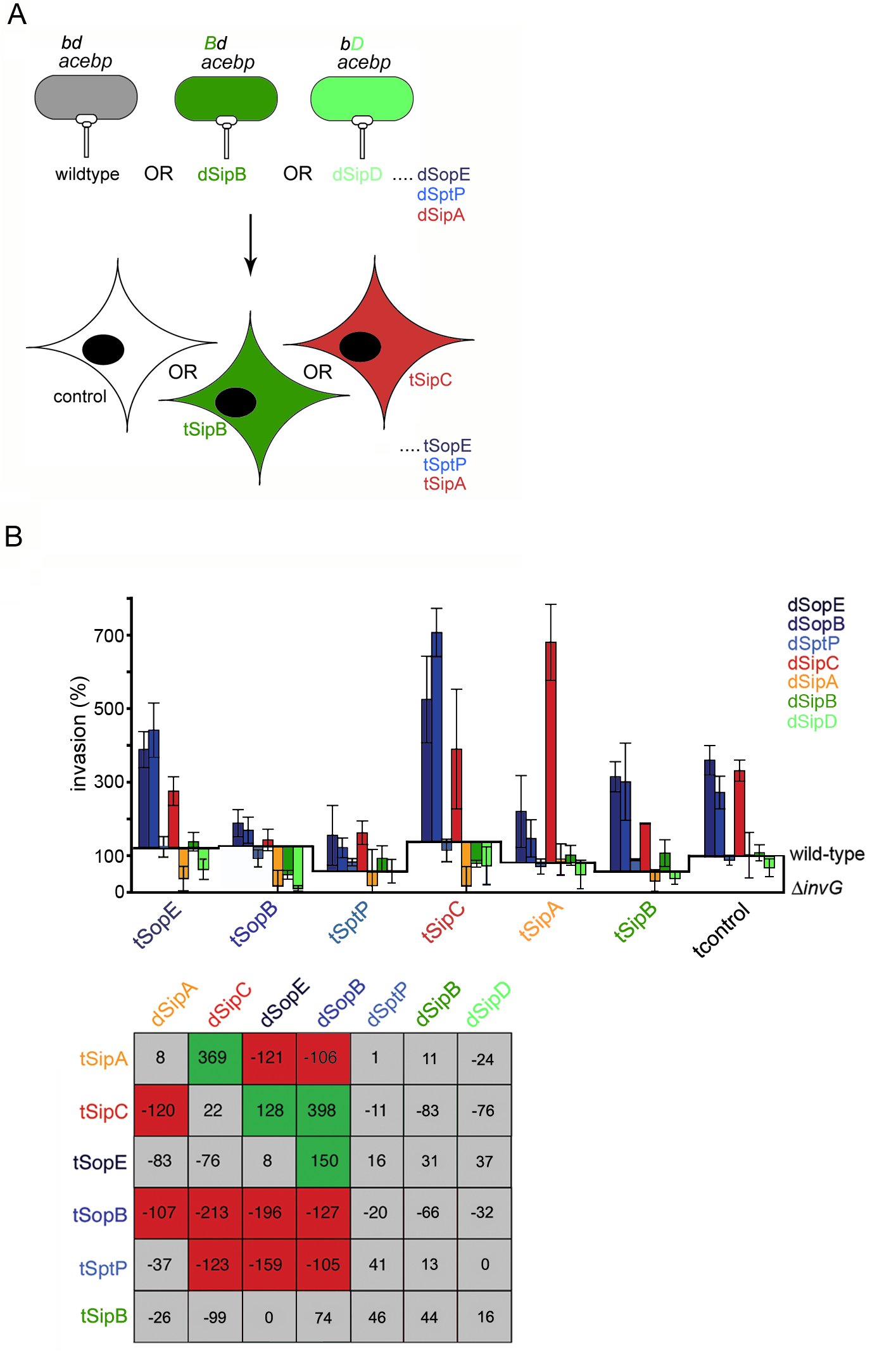

Supplement: Figure S3 — The T3SS translocator SipB and SipD are not invasion effectors. A. Schematic illustrating trans BENEFIT screening (the infection of cells expressing individual entry effectors by wild-type WT or effector-augmented S.typhimurium strains). Wild-type (WT) bacteria endogenously express, secrete and deliver sipA, sipC, sopE, sopB and sptP (abbreviated to acebp) and sipB and sipD (abbreviated separately as bd). Effector-augmented strains each express, secrete and deliver mildly increased levels of an individual plasmid-encoded effector in the WT background [enhanced effector shown in capitals, e.g. acebp Bd (dSipB) and acebp bD (dSipD) produce increased levels of SipB and SipD, respectively]. Cultured cells were transfected with individual entry effectors (denoted t-effector) prior to infection. B. Upper: Cultured fibroblasts were transfected (t) with individual effectors prior to infection with WT or effector-augmented (d-effector) S.typhimurium strains. Invasion rates after 60 min were compared to WT (assigned as 100%). Results are mean±SEM of 4 independent experiments each performed in triplicate. Baselines ‘wild-type’ and ‘ΔinvG’ denote S.typhimurium SL1344 and S.typhimurium ΔinvG (T3SS deficient) invasion in each transfectant background, respectively. Lower: Table showing differences in invasion rates (%) after correction. Shading denotes a significant increase (green), significant decrease (red) or no significant change (grey) in invasion (Mann Whitney U p<0.05). (9.10 MB TIF) [file ppat.1000037.s003.tif]

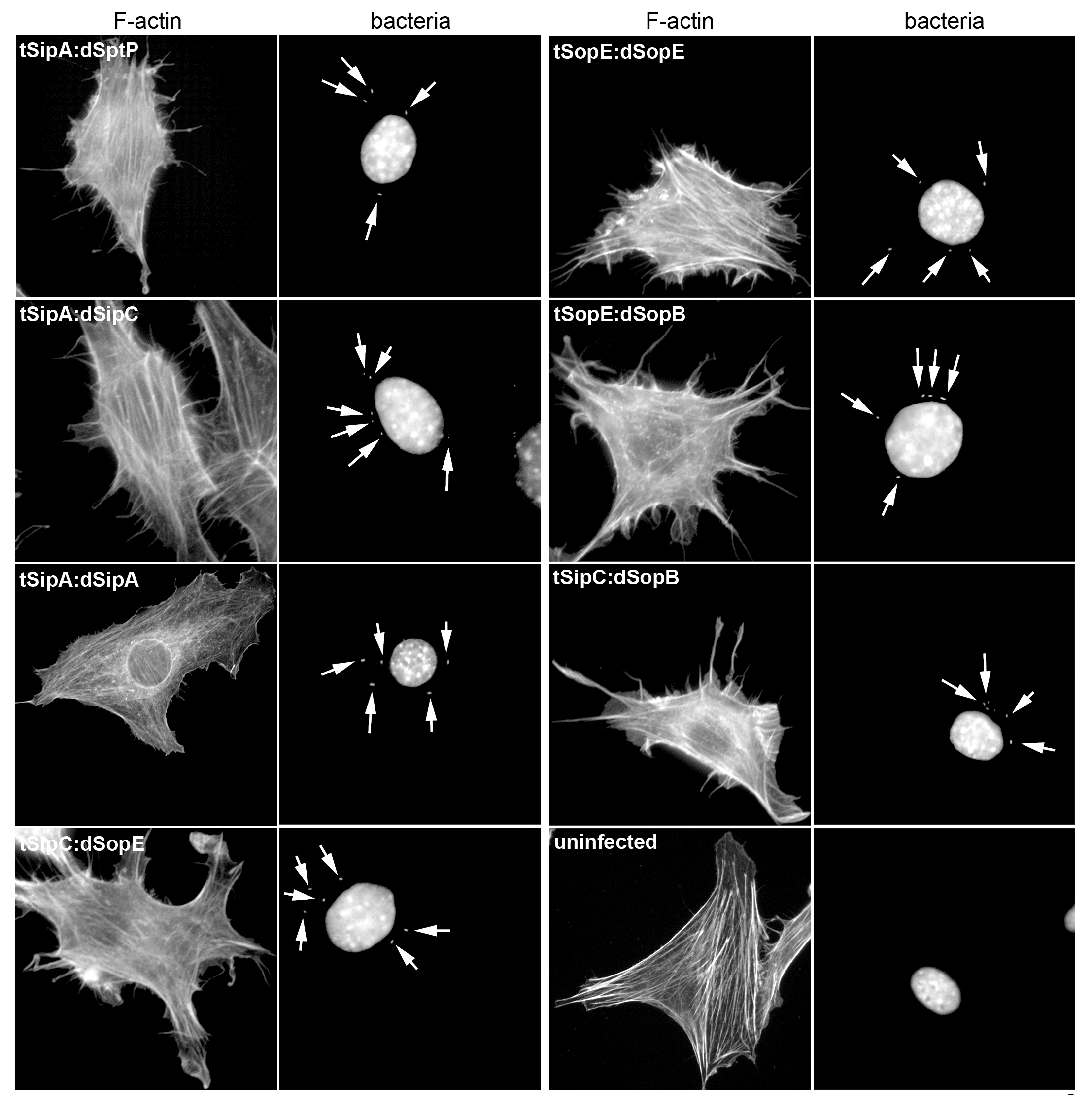

Supplement: Figure S4 — Actin rearrangements induced during infection of transfected cells. Cultured fibroblasts were transfected with indicated effectors (t-effector) and subsequently infected with effector-augmented (d-effector) S.typhimurium strains. Cells were fixed 60 min post infection and double fluorescence stained to visualise F-actin and bacteria/cell nuclei. Arrows indicate internalized bacteria. (1.66 MB TIF) [file ppat.1000037.s004.tif]

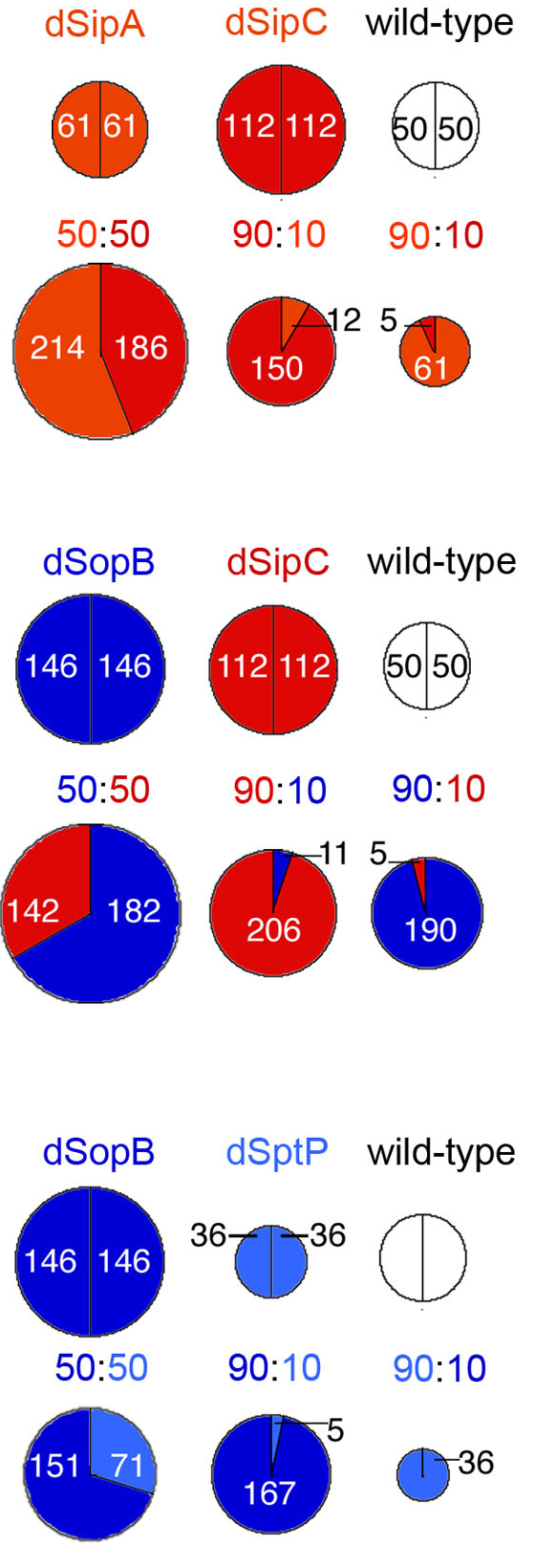

Supplement: Figure S5 — Effector synergy is abolished by biasing the relative levels of each strain. S.typhimurium SL1344 or effector augmented (d-effector) strains were mixed pair wise (MOI 50) at 50∶50, 90:10 and 10:90 ratios. Invasion of each strain was assessed using selectable markers after 60 min (Figure 4A). Results are the mean of four independent experiments each performed in triplicate. Pie charts depict total invasion by each combination (size; combined %) and relative contribution of each strain (division; %) using the indicated ratios. (0.69 MB TIF) [file ppat.1000037.s005.tif]
